# Neighborhood Social Cohesion and Sleep Health by Age, Sex/Gender, and Race/Ethnicity in the United States

**DOI:** 10.3390/ijerph17249475

**Published:** 2020-12-17

**Authors:** Dana M. Alhasan, Symielle A. Gaston, W. Braxton Jackson, Patrice C. Williams, Ichiro Kawachi, Chandra L. Jackson

**Affiliations:** 1 Epidemiology Branch, National Institute of Environmental Health Sciences, National Institute of Health, Department of Health and Human Services, Research Triangle Park, Durham, NC 27709, USA; dana.alhasan@nih.gov (D.M.A.); symielle.gaston@nih.gov (S.A.G.); patrice.williams@nih.gov (P.C.W.); 2 Social & Scientific Systems, Inc., Durham, NC 27703, USA; braxton.jackson@dlhcorp.com; 3 Department of Social and Behavioral Sciences, Harvard T.H. Chan School of Public Health, Boston, MA 02115, USA; ikawachi@hsph.harvard.edu; 4 Intramural Program, National Institute on Minority Health and Health Disparities, National Institutes of Health, Department of Health and Human Services, Bethesda, MD 20814, USA

**Keywords:** residence characteristics, community support, social support, sleep, sleep initiation and maintenance disorders, African Americans, Hispanic Americans, minority groups

## Abstract

Although low neighborhood social cohesion (nSC) has been linked with poor sleep, studies of racially/ethnically diverse participants using multiple sleep dimensions remain sparse. Using National Health Interview Survey data, we examined overall, age, sex/gender, and racial/ethnic-specific associations between nSC and sleep health among 167,153 adults. Self-reported nSC was categorized into low, medium, and high. Very short sleep duration was defined as <6 h; short as <7 h, recommended as 7–9 h, and long as ≥9 h. Sleep disturbances were assessed based on trouble falling and staying asleep, waking up feeling unrested, and using sleep medication (all ≥3 days/times in the previous week). Adjusting for sociodemographics and other confounders, we used Poisson regression with robust variance to estimate prevalence ratios (PRs) and 95% confidence intervals (CIs) for sleep dimensions by low and medium vs. high nSC. The mean age of the sample was 47 ± 0.1 years, 52% of those included were women, and 69% were Non-Hispanic (NH)-White. Low vs. high nSC was associated with a higher prevalence of very short sleep (PR = 1.29; (95% CI = 1.23–1.36)). After adjustment, low vs. high nSC was associated with very short sleep duration among NH-White (PR = 1.34 (95% CI = 1.26–1.43)) and NH-Black (PR = 1.14 (95% CI = 1.02–1.28)) adults. Low nSC was associated with shorter sleep duration and sleep disturbances.

## 1. Introduction

Short sleep duration and sleep disturbances, such as trouble falling asleep, are highly prevalent among the United States (U.S.) population [1]. For instance, it is estimated that one-third of adults habitually obtain less than the recommended amount of at least seven hours of sleep [2]. Groups even more likely to experience short sleep and sleep disturbances include those ≥50 years old compared to 18–30 years old [3], women compared to men [4], and non-Hispanic (NH)-Black [2,5,6] Hispanic/Latinx [5,7], and Asian [8] compared to NH-White adults. For example, one study reported that 43.4% of NH-Black, 31.5% of Hispanic/Latinx, and 27.1% of Asians obtained less than seven hours of sleep, compared to 19.4% of NH-White adults [5]. It is important to identify factors that may influence sleep health because a short sleep duration and sleep disturbances have been shown to increase the risk of a variety of chronic health conditions, such as hypertension, type 2 diabetes, and cardiovascular disease [9], that contribute to the leading causes of death in the U.S. 

Recent studies have found that the neighborhood environment may be related to sleep health [10,11], which offers a potential point of intervention. Neighborhood social cohesion (nSC), or the degree of connectedness and solidarity among people in a community [12], is hypothesized to positively influence sleep health by creating support networks, increasing perceived safety, and reinforcing social norms such as not staying up late [13,14]. Strong support networks and feelings of safety may relieve stress and contribute to better mental health and thus positively influence sleep. Conversely, less cohesive neighborhoods are hypothesized to negatively influence sleep health by increasing vigilance, social isolation, and physical inactivity [15]. The subsequent activation of stress pathways may elicit the chronic activation of the body’s central stress response system, the hypothalamus–pituitary–adrenal axis, as recently demonstrated by a systematic review [16], thus negatively influencing sleep [17]. 

The nSC–sleep relationship may differ by age, sex/gender, and race/ethnicity. For instance, older adults who are more reliant on their immediate surroundings [18] may be more influenced by the social neighborhood environment compared to more mobile middle-aged and young adults [19,20]. While no study, to our knowledge, has reported sex/gender-specific associations between nSC and sleep, women compared to men may be more sensitive to and more influenced by certain social factors, including the strength of support networks, perception of neighborhood safety, and influence of social norms, thus impacting their perception of nSC [21,22]. nSC may also vary by race/ethnicity, as previously demonstrated [23,24]. Stemming from residential segregation, racial/ethnic minority groups are generally more likely to experience adverse neighborhood environments, which may compromise social cohesion among neighbors [25,26]. Therefore, it is important to explicitly study how the neighborhood environment may contribute to sleep disparities [27] in racial/ethnic minority groups [17,28]. Further, no study, to our knowledge, has examined the nSC–sleep relationship at the intersection of age, sex/gender, and race/ethnicity. Since all three social categories may modify the influence of nSC on sleep health, it is important to examine the potential synergy or compounding relationship between nSC and age, sex/gender, and race/ethnicity.

To address these gaps in the literature, we aimed to use data from the National Health Interview Survey to estimate overall, age, sex/gender, and racial/ethnic-specific cross-sectional associations between nSC and multiple sleep dimensions. We hypothesized that the perception of living in a neighborhood with low vs. high and medium vs. high social cohesion would be associated with shorter sleep duration and more sleep disturbances (e.g., insomnia symptoms) among all participants and that the magnitude of the association would be stronger for ≥50 year-old participants, women, and racial/ethnic minority groups. We also hypothesized that the associations between nSC and sleep dimensions would be stronger among women of racial/ethnic minority groups who are ≥50 years old compared to NH-White women who are ≥50 years old. Lastly, we hypothesized that the associations between nSC and sleep dimensions would be stronger among men of racial/ethnic minority groups who are ≥50 years old compared to NH-White men who are ≥50 years old.

## 2. Materials and Methods 

### 2.1. National Health Interview Survey

Participant data for this study came from the National Health Interview Survey (NHIS), from which survey years from 2013 to 2018 were retrieved by the Integrated Health Interview Series [29]. The NHIS is a series of annual, cross-sectional, household surveys conducted via computer-assisted in-person interviews among the non-institutionalized U.S. adult population. Trained interviewers obtained information regarding medical conditions, health care access, and health behaviors for each member of the sampled household. The NHIS uses a three-stage stratified cluster probability sampling design to obtain a nationally representative sample. A randomly selected adult and child (if present, although not included in the current analysis) from each household provided more specific health-related information. A detailed description of the NHIS procedures has been previously published [30]. The response rate for sample adults was 56.1% (range: 61.2% (2013) to 53.1% (2018)). Sampling weights were used to account for the survey’s complex sampling design, non-response, and the oversampling of certain groups (e.g., racial/ethnic minorities; older adults), which resulted in unequal probabilities of selection. Each study participant provided informed consent to the NHIS, and the National Institute of Environmental Health Sciences’ Institutional Review Board waived approval for publicly available, secondary data with no identifiable information. 

### 2.2. Study Population

Participants (≥18 years of age) from all 50 states and the District of Columbia were included in the sample. Of the 190,113 participants, those with missing or implausible data for key variables, such as race/ethnicity (n = 4348), sleep duration (n = 5986), sleep disturbances (n = 1618), and nSC (n = 9675), were excluded, and those of Native American race (n = 1333) were excluded due to the small sample size. The final analytical sample size was 167,153 participants (Supplemental Figure S1).

### 2.3. Exposure Assessment: Neighborhood Social Cohesion

nSC was measured using a modified version of a four-item scale developed by the Project on Human Development in Chicago Neighborhoods Community Survey [31]. Participants responded on a Likert scale (1 = definitely agree; 2 = somewhat agree; 3 = somewhat disagree; and 4 = definitely disagree) to the following four statements: (1) “People in this neighborhood help each other out”; (2) “There are people I can count on in this neighborhood”; (3) “People in this neighborhood can be trusted”; and (4) “This is a close-knit neighborhood”. Responses were reverse-coded, and the nSC variable was calculated as the sum of the four response options. Scores ranged from 4–16, with higher scores indicating greater perceived levels of nSC. nSC scores were further categorized into three groups based on previous literature [32]: low (<12), medium (12–14), and high (≥15). 

### 2.4. Outcome Assessment: Self-Reported Sleep Duration and Sleep Disturbances

Sleep duration was measured by asking participants, “On average, how many hours of sleep do you get in a 24 h period?”. Interviewers reported sleep hours in whole numbers as well as rounded values of ≥30 min up to the nearest hour and rounded values <30 min down to the nearest hour. Responses were categorized as very short (<6 h), short (<7 h), recommended (7–9 h) or long (>9 h), based upon the National Sleep Foundation categories [33]. Very short and short sleep were not mutually exclusive categories.

Sleep disturbances were measured by asking participants the following four questions: (1) “In the past week, how many times did you have trouble falling asleep?”; (2) “In the past week, how many times did you have trouble staying asleep?”; (3) “In the past week, on how many days did you wake up feeling well rested?”; and (4) “In the past week, how many times did you take medication to help you fall asleep or stay asleep?”. If participants reported experiencing a sleep disturbance for ≥3 days/times per week (i.e., trouble falling asleep, trouble staying asleep, waking up feeling unrested, or taking medication to help fall asleep), they were considered as having a sleep disturbance. Assessed in combination and separately, insomnia symptoms included reports of either trouble falling asleep ≥3 vs. <3 times/week and/or difficulty maintaining sleep ≥3 vs. <3 times/week.

### 2.5. Potential Confounders

Potential sociodemographic confounders included age (18–30, 31–49, and ≥50 years), sex/gender (women or men), race/ethnicity (NH-White, NH-Black, Hispanic/Latinx, and Asian), educational attainment (<high school, high school graduate, some college, and above college-level), annual household income (<$35,000, $35,000–$74,999, and ≥$75,000), employment status (employed in the labor force or not), occupational class (professional/management, support services, or laborers), region of residence (Northeast, Midwest, South, and West), and marital status (married/living with partner/cohabitating, divorced/widowed/separated, or single/no live-in partner). Potential health behavior confounders included smoking status (never, former, or current), alcohol consumption (never, former, or current), and leisure-time physical activity based on weekly minutes of engagement categorized as never/unable, meets physical activity guidelines, or does not meet physical activity guidelines [34]. Potential clinical characteristic confounders included general health status (excellent/very good, good, or fair/poor); serious mental illness, defined as a score ≥13 on the Kessler-6 psychological distress scale [35] (yes or no); body mass index (BMI), calculated by dividing respondents’ self-reported weight in kilograms by their self-reported height in meters squared and categorized as recommended (18.5–< 25 kg/m^2^), overweight (25–29.9 kg/m^2^), and obese (≥30 kg/m^2^); and healthcare provider diagnosis of dyslipidemia, hypertension, and diabetes/prediabetes (yes or no). Rather than considering each health behavior and clinical characteristic separately, a dichotomized measure of “ideal” cardiovascular health was estimated based on meeting all of the following criteria: never smoked or former smoker (including quitting smoking >12 months prior to interview); met physical activity guidelines; recommended BMI; and reported no prior diagnosis of dyslipidemia, hypertension, or diabetes/prediabetes [36].

### 2.6. Potential Modifiers: Age, Sex/Gender, and Race/Ethnicity

Participants self-identified their age, sex/gender, and race/ethnicity. Age was categorized as 18–30, 31–49, and ≥50 years. Sex/gender, assessed in a binary vs. non-binary manner, was dichotomized as women versus men. Race/ethnicity was categorized as NH-White alone, NH-Black alone, Hispanic/Latinx (of any race), and Asian. 

### 2.7. Statistical Analyses

Descriptive statistics were computed; continuous variables were presented as means ± standard errors (S.E.), and categorical variables were presented as weighted percentages after applying direct standardization using the 2010 U.S. Census population. We compared the three levels of nSC across sociodemographic, health behavior, and clinical characteristics for all participants. 

To test associations between nSC and sleep dimensions, we used Poisson regression with robust variance to directly estimate prevalence ratios [37] (PRs) and 95% confidence intervals (CIs) of nSC for each sleep dimension overall, by age, sex/gender, and race/ethnicity, and age–sex/gender–race/ethnicity. This model with adjusted variances has been shown to provide accurate point and interval estimates using either count or binary data during one cross-sectional point in time. Furthermore, this model directly estimates PRs, unlike a logistic regression model, which provides estimated odds ratios that overestimate associations with outcomes of high prevalence, such as poor sleep health. PRs are also easier to communicate and are more interpretable than odds ratios. The overall model was statistically adjusted for the following confounders: age, sex/gender, race/ethnicity, educational attainment, annual household income, employment status, occupational class, region of residence, marital status, alcohol consumption, health status, serious mental illness, and “ideal” cardiovascular health. To test for differences by age, sex/gender, and race/ethnicity, separately and together, respective interaction terms (e.g., nSC * age) were added to the overall model. Analyses were conducted in SAS version 9.4 for Windows (Cary, North Carolina), and a two-sided *p*-value of 0.05 was used to determine statistical significance. 

## 3. Results

### 3.1. Study Population Characteristics

Among 167,153 participants, 32% (n = 53,364), 33% (n = 55,163), and 35% (n = 58,626) perceived their neighborhoods to have low, medium, and high social cohesion, respectively (Table 1). The mean age was 47.4 ± 0.1 years. Approximately 51.9% were women and 69.2% self-identified as NH-White, 11.1% as NH-Black, 14.2% as Hispanic/Latinx, and 5.5% as Asian. Most participants reported obtaining the recommended sleep duration (64.7%). The most frequently reported sleep disturbance was waking up feeling unrested (42.8%), followed by insomnia symptoms (33.0%), trouble staying asleep (27.4%), trouble falling asleep (19.9%), and using sleep medications to help fall asleep (9.8%). 

A higher percentage of those aged 18–30 years old (18.7%) lived in a neighborhood with low compared to medium (16.2%) and high (13.7%) social cohesion, while a higher percentage of those aged 31–49 years old (26.0%) lived in a neighborhood with high compared to medium (23.5%) and low (21.0%) social cohesion. A higher percentage of NH-Black (14.1%) and Hispanic/Latinx (19.0%) participants lived in a neighborhood with low social cohesion compared to medium (11.7% and 13.9%, respectively) and high (7.9% and 10.2%, respectively) social cohesion, while a higher percentage of NH-White respondents lived in neighborhood with high (77.0%) compared to medium (68.1%) and low (61.7%) social cohesion. Overall, the prevalence of very short sleep and short sleep was higher among those who reported living in a neighborhood with low (11.8% and 36.2%, respectively) compared to medium (7.9% and 30.5%) and high (7.5% and 27.9%) social cohesion. The prevalence of sleep disturbances was also higher among those living in a neighborhood with low compared to medium and high social cohesion. For example, the prevalence of trouble staying asleep was also higher among those who reported living in a neighborhood with low (32.2%) compared to medium (26.3%) and high (24.4%) social cohesion (Table 1). Additional sociodemographic characteristics by age, sex/gender, and race/ethnicity are described in Supplemental Tables S1–S4.

### 3.2. Neighborhood Social Cohesion and Multiple Sleep Dimensions 

**Figure 1:**
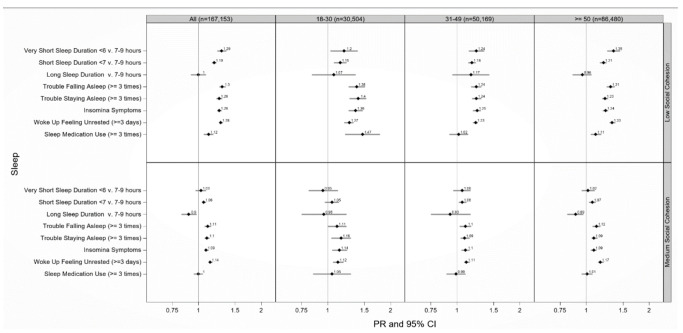
Adjusted prevalence ratios of sleep health by low and medium compared to high neighborhood social cohesion among 18–30, 31–49, and ≥50 year-old respondents to the National Health Interview Survey, 2013–2018 (N = 167,153). PR = prevalence ratio; CI = confidence interval. Models adjusted for sex/gender (women or men), educational attainment (<high school, high school graduate, some college, ≥college), annual household income (<$35,000, $35,000–$74,999, $75,000+), occupational class (professional/management, support services, laborers), region of residence (Northeast, Midwest, South, West), alcohol consumption (never, former, current), serious mental illness (Kessler-6 psychological distress scale score ≥ 13), “ideal” cardiovascular health (never smoking/quit >12 months prior to interview, BMI 18.5 to <25 kg/m^2^, meeting physical activity guidelines, and no prior diagnosis of dyslipidemia, hypertension, or diabetes/prediabetes), marital/co-habiting status (married/living with partner or cohabitating, divorced/widowed/separated, single/no live-in partner), employment status (unemployed, employed), and health status (excellent/very good, good, fair/poor). All models were additionally adjusted for age (18–30, 31–49, 50+ years). Reference level: high neighborhood cohesion Note. All estimates were weighted for the survey’s complex sampling design. Insomnia symptoms defined as either trouble falling asleep and/or difficulty maintaining sleep 3+ times a week.

Participants who lived in a neighborhood with low vs. high social cohesion had on average 29% (PR = 1.29 (95% CI: 1.23–1.36)) higher prevalence of very short sleep and 19% (PR = 1.19 (95% CI: 1.16–1.22)) higher prevalence of short sleep duration, after adjustment (Figure 1). Participants who reported living in a neighborhood with medium vs. high social cohesion also experienced a higher prevalence of very short (PR = 1.03 (95% CI: 0.97–1.09)) and short sleep duration (PR = 1.06 (95% CI: 1.04–1.09)), after adjustment. Participants who reported living in a neighborhood with low vs. high social cohesion also experienced a higher prevalence of trouble staying asleep (PR = 1.26 (95% CI: 1.22–1.30)) and insomnia (PR = 1.26 (95% CI: 1.23–1.29)), after adjustment. Similar patterns emerged between low vs. high and medium vs. high nSC with other sleep disturbances (e.g., trouble falling asleep), after adjustment (Figure 1).

### 3.3. Neighborhood Social Cohesion and Multiple Sleep Dimensions by Age

Low vs. high nSC was associated with very short sleep duration among ≥50 (PR = 1.35 (95% CI: 1.26–1.45)), 31–49 (PR = 1.24 (95% CI: 1.14–1.35)) and 18–30 year-old respondents (PR = 1.20 (95% CI: 1.03–1.39)), after adjustment (Figure 1). Those aged 18–30 years old living in neighborhoods with low vs. high nSC had 40% (PR = 1.40 (95% CI: 1.27–1.53)) more trouble staying asleep and 47% (PR = 1.47 (95% CI: 1.21–1.78)) higher use of sleep medications, while those aged ≥ 50 years old had 23% (PR = 1.23 (95% CI: 1.19–1.27)) more trouble staying asleep and 11% (PR = 1.11 (95% CI: 1.05–1.18)) more use of sleep medications, after adjustment (Figure 1).

### 3.4. Neighborhood Social Cohesion and Multiple Sleep Dimensions by Sex/Gender

**Figure 2:**
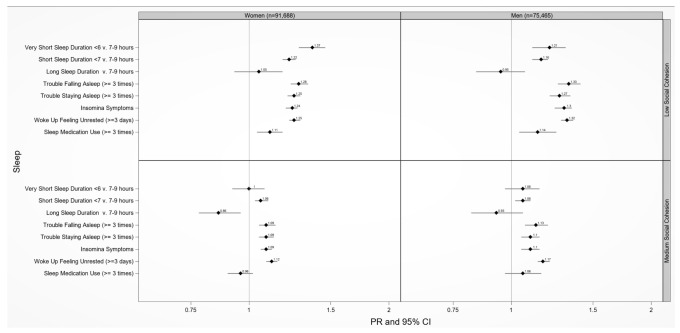
Adjusted prevalence ratios of sleep health by low and medium compared to high neighborhood social cohesion among U.S. women and men, National Health Interview Survey, 2013–2018 (N = 167,153). Sex/gender specific models adjusted for age (18–30, 31–49, 50+ years), race/ethnicity (NH-White, NH-Black, Hispanic, and Asian), educational attainment (<high school, high school graduate, some college, ≥college), annual household income (<$35,000, $35,000–$74,999, $75,000+), occupational class (professional/management, support services, laborers), region of residence (Northeast, Midwest, South, West), alcohol consumption (never, former, current), serious mental illness (Kessler-6 psychological distress scale score ≥ 13), “ideal” cardiovascular health (never smoking/quit > 12 months prior to interview, BMI 18.5 to <25 kg/m^2^, meeting physical activity guidelines, and no prior diagnosis of dyslipidemia, hypertension, or diabetes/prediabetes), marital/co-habiting status (married/living with partner or cohabitating, divorced/widowed/separated, single/no live-in partner), employment status (unemployed, employed), and health status (excellent/very good, good, fair/poor). Reference level: high neighborhood cohesion. Note. All estimates are weighted for the survey’s complex sampling design. Insomnia symptoms defined as either trouble falling asleep and/or difficulty maintaining sleep 3+ times a week.

Women and men who lived in a neighborhood with low vs. high social cohesion had 37% (PR = 1.37 (95% CI: 1.28–1.46)) and 21% (PR = 1.21 (95% CI: 1.11–1.31)) higher prevalence of very short sleep duration, respectively, after adjustment (Figure 2). Women and men who lived in a neighborhood with low vs. high social cohesion had 22% (PR = 1.22 (95% CI: 1.18–1.26)) and 16% (PR = 1.16 (95% CI: 1.11–1.20)) higher prevalence of short sleep duration, respectively, after adjustment. Low vs. high nSC was associated with waking up feeling unrested among women (PR = 1.25 (95% CI: 1.22–1.29)) and men (PR = 1.32 (95% CI: 1.28–1.36)). Medium vs. high nSC was associated with waking up feeling unrested among women (PR = 1.12 (95% CI: 1.09–1.15)) and men (PR = 1.17 (95% CI: 1.14–1.21)) (Figure 2).

### 3.5. Neighborhood Social Cohesion and Multiple Sleep Dimensions by Race/Ethnicity

**Figure 3:**
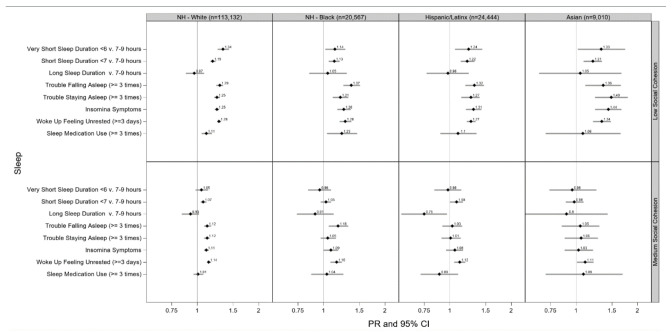
Adjusted prevalence ratios of sleep health by low and medium compared to high neighborhood social cohesion among U.S. racial/ethnic adults, National Health Interview Survey, 2013–2018 (N = 167,153). Race/ethnic-specific model adjusted for age (18–30, 31–49, 50+ years), sex/gender (women or men), educational attainment (<high school, high school graduate, some college, ≥college), annual household income (<$35,000, $35,000–$74,999, $75,000+), occupational class (professional/management, support services, laborers), region of residence (Northeast, Midwest, South, West), alcohol consumption (never, former, current), serious mental illness (Kessler-6 psychological distress scale score ≥13), “ideal” cardiovascular health (never smoking/quit >12 months prior to interview, BMI 18.5 to <25 kg/m^2^, meeting physical activity guidelines, and no prior diagnosis of dyslipidemia, hypertension, or diabetes/prediabetes), marital/co-habiting status (married/living with partner or cohabitating, divorced/widowed/separated, single/no live-in partner), employment status (unemployed, employed), and health status (excellent/very good, good, fair/poor). Reference level: high neighborhood cohesion. Note. All estimates are weighted for the survey’s complex sampling design. Insomnia symptoms defined as either trouble falling asleep and/or difficulty maintaining sleep 3+ times a week.

Low vs. high nSC was associated with very short sleep duration among NH-White (PR = 1.34 (95% CI: 1.26–1.43)), Asian (PR = 1.33 (95% CI: 1.02–1.74)), Hispanic/Latinx (PR = 1.24 (95% CI: 1.06–1.44)), and NH-Black (PR = 1.14 (95% CI: 1.02–1.28)) adults, after adjustment (Figure 3). Likewise, low vs. high nSC was associated with short sleep duration among Asians (PR = 1.21 (95% CI: 1.09–1.34)), Hispanic/Latinx (PR = 1.22 (95% CI: 1.13–1.32)), NH-White (PR = 1.19 (95% CI: 1.16–1.23)), and NH-Black (PR = 1.13 (95% CI: 1.06–1.20)) adults, after adjustment. Asian adults who lived in a neighborhood with low vs. high nSC had 49% more trouble staying asleep (PR = 1.49 (95% CI: 1.24–1.79)) and 44% (PR = 1.44 (95% CI: 1.24–1.67)) more insomnia symptoms, after adjustment. NH-Black adults who lived in a neighborhood with low vs. high nSC used sleep medications 23% (PR = 1.23 (95% CI: 1.04–1.46)) more, after adjustment. Similar patterns emerged between medium vs. high nSC with sleep duration among racial/ethnic groups although mostly non-significant (Figure 3).

### 3.6. Neighborhood Social Cohesion and Multiple Sleep Dimensions by Age, Sex/Gender, and Race/Ethnicity

Low vs. high nSC was associated with very short sleep duration among NH-Black (PR = 1.79 (95% CI: 1.15–2.79)) and Asian (PR = 1.57 (95% CI: 0.57–4.31)) women 18–30 years old, after adjustment (Table 2). Low vs. high nSC was associated with very short sleep duration among Asian (PR = 1.81 (95% CI: 1.15–2.85)), Hispanic/Latinx (PR = 1.53 (95% CI: 1.19–1.97)), and NH-White (PR = 1.40 (95% CI: 1.25–1.56)) women ≥ 50 years old, after adjustment. Low vs. high nSC was associated with more trouble falling asleep among Hispanic/Latinx women 18–30 years old (PR = 1.42 (95% CI: 1.03–1.94)), after adjustment. Among NH-Black women ≥50 years old, those who lived in neighborhoods with low vs. high social cohesion had 20% (PR = 1.20 (95% CI: 1.07–1.36)) higher prevalence of insomnia symptoms, after adjustment.

Low vs. high nSC was associated with short sleep duration (PR = 1.61 (95% CI: 1.19–2.17)), trouble falling asleep (PR = 3.77 (95% CI: 1.97–7.22)), trouble staying asleep (PR = 3.16 (95% CI: 1.83–5.47)), and insomnia symptoms (PR = 2.61 (95% CI: 1.59–4.31)) among NH-Black men 18–30 years old, after adjustment (Table 2).

## 4. Discussion

In this large sample of U.S. adults, we found that perceived neighborhood social cohesion was associated with sleep health. Consistent with our hypothesis, we found that participants who reported living in a neighborhood with low vs. high social cohesion generally experienced shorter sleep duration and more sleep disturbances. Furthermore, also consistent with our hypothesis, we found important modifications of the nSC–sleep relationship by age, sex/gender, and race/ethnicity separately as well as together, although the strength of the interactions was not always in the expected direction. For instance, the interactions between nSC, sleep disturbances (e.g., trouble staying asleep) and age were stronger among younger (18–30 years of age) than older adults (≥50 years of age), except waking up feeling unrested. We observed stronger associations of sleep duration (e.g., very short sleep) among women living in areas with low vs. high nSC compared to men, which corresponded with our hypothesis, although no other notable differences were observed between sex/gender except for the fact that waking up feeling unrested was stronger among men than women. We also observed stronger associations between low vs. high nSC and very short sleep duration in NH-White adults compared to NH-Black adults, while associations for sleep disturbances (e.g., insomnia) were stronger among Asian adults, except for the fact that the use of sleep medications was stronger among NH-Black adults.

Our overall findings that those living in a neighborhood with lower social cohesion experienced shorter sleep duration and more sleep disturbances are supported by previous studies [10,20,32], including studies with objective sleep measures [17]. As an example, our finding that participants ≥50 years old living in a neighborhood with lower social cohesion experienced shorter sleep duration is supported by previous studies [10,17,20]. For instance, a study using data from the Health Retirement Survey found that lower nSC was associated with higher odds of trouble falling asleep among those ≥50 years old [20]. Older adults may be more influenced by the social neighborhood environment compared to middle-aged and young adults considering a potentially reduced network size due, for instance, to retirement, death of loved ones, and compromised health [19]. Despite these consistent findings with prior work, an interesting, novel finding we observed was stronger associations between low vs. high nSC and sleep disturbances, such as that difficulties staying asleep were stronger in younger (18–30 years old) compared to older adults (≥50 years old). While there are no other studies to compare these results with, these findings may indicate the need to intervene in sleep earlier in life, which will likely benefit overall health. 

We also found that correlations between low vs. high nSC and shorter sleep duration were stronger in women than men. This is consistent with the idea that women may be more influenced by their neighborhood environment compared to men [38]. Based on a socioecological theory, prior work suggests that women’s greater vulnerability to the social neighborhood environment is due to differences in how women are impacted by support networks, how they perceive their environment, and the types of stressors women face on a daily basis, particularly in terms of the social roles women occupy [22,39,40]. Women may leverage social cohesion when engaging in physical activity and other healthy behaviors that can positively influence sleep. Men, on the other hand, may engage in social activities that do not require social cohesion. In fact, prior work examining other aspects of the social neighborhood environment, such as safety, and sleep dimensions also found stronger associations among women compared to men, which is consistent with our findings [38,41,42]. Nonetheless, prior work specifically examining nSC and sleep dimensions did not find modification by sex/gender [10,43]. We also did not observe variations in associations with sleep disturbances by sex/gender, except for the fact that waking up feeling unrested was stronger among men than women. Given these mixed findings, further studies are needed to determine if these effect modification results are replicable and, if so, to assess potential drivers.

Another finding of our work was the potential effect modification of low vs. high nSC and sleep dimensions by race/ethnicity. Although these findings need to be interpreted with caution due to the substantial overlap in the CIs observed, our results suggest that the impact of lower nSC on multiple sleep dimensions may impact racial/ethnic groups differently. For example, the impact of lower nSC on short sleep duration could be larger among NH-White adults, the impact of lower nSC on trouble staying asleep could be larger among Asian adults, and the impact of lower nSC on use of sleep medication could be larger among NH-Black adults. While the potential determinants driving these differences is unclear, these results add to a growing body of literature demonstrating the relationship between low vs. high nSC and poor sleep health among racial/ethnic minority groups (i.e., NH-Black, Hispanic/Latinx, and Asian adults) [10,17,32,44]. It is noteworthy, however, that our findings were not consistent with two previous studies, which may be attributed to a difference in the operationalization and/or modeling of sleep dimensions. For example, findings from the Jackson Heart Study did not find an association between nSC and sleep disturbances after adjustment among a NH-Black population [43]. Their dichotomization of sleep disturbances compared to our ordinal operationalization may not capture meaningful differences in the average number of days of sleep disturbances. Another study that did not find an association between nSC and sleep health among a NH-Black population modeled sleep duration in a linear regression [45]; this does not account for the non-normal distribution of sleep duration. Rather, non-parametric methods, such as a Poisson regression, can better model the natural logarithm of average hours of sleep.

Our study is the first, to our knowledge, to examine the relationship between nSC and sleep health by age–sex/gender–race/ethnic groups. Our findings suggest that multiple social categories intersect to influence sleep health, although the findings were inconsistent with our hypotheses that associations would be stronger among racial/ethnic minority women and men ≥50 years old compared to NH-White women and men ≥50 years old. For example, we observed that NH-White women ≥50 years old and NH-Black women 18–30 years old who lived in neighborhoods with lower social cohesion experienced shorter sleep duration. We also observed that NH-Black women ≥50 years old who lived in neighborhoods with lower social cohesion experienced more insomnia symptoms, while Hispanic/Latinx women 18–30 years old who lived in neighborhoods with lower social cohesion experienced more trouble falling asleep. Additionally, we observed NH-Black 18–30-year-old men living in low vs. high nSC experienced, on average, greater short sleep duration and more sleep disturbances (e.g., insomnia symptoms). Our findings are similar to another study that found nSC–sex/gender–race interactions with inflammatory biomarkers [46], which are associated with more sleep disturbances [47]. While our observed measures of association were not strikingly different, perhaps due to our large sample size, our findings suggest that sleep disparities may be explained by the impact of neighborhood environments across multiple identities and that age, sex/gender, and race/ethnicity impact each other in such a way that one identity alone cannot explain the sleep disparities without the intersection of the other identities. 

It is hypothesized that nSC, and neighborhood environments in general, influence sleep health through different mechanisms including psychosocial, physiological, and social engagement pathways. Residing in neighborhoods with lower social cohesion and adverse environments characterized by discrimination, environmental hazards, and violence may increase anxiety, depression, and stress [15]. This may then lead to the dysregulation of the hypothalamic–pituitary–adrenal axis that impacts biological rhythms and sleep [15]. Similarly, adverse environments may also increase allostatic load and inflammatory biomarkers [12], which in turn impact sleep health. Neighborhood environments may also influence sleep health via social engagement, such as sharing resources, facilitating access to health related information, providing tangible support (e.g., transportation), reinforcing social norms for behaviors, and enhancing self-efficacy [48]. These hypothesized mechanisms may also differ by social categories. Older adults with limited mobility spend more time in their immediate neighborhood environments and thus rely more on their surroundings [18]. Women are thought to be more impacted by their social environment compared to men [22], and women may perceive neighbors’ connectedness differently and utilize social support more than men. Even in neighborhoods that are limited in resources, linguistic and cultural similarities may allow for racial/ethnic enclaves to supplement smaller social networks to address the ongoing needs of those experiencing poor sleep [23]. Without a doubt, the intersection of these social categories, such as older NH-Black women, will be impacted by nSC via different mechanisms.

The limitations of this study include its cross-sectional design, which prevents causal inference regarding nSC and sleep health. As such, reverse causation is possible, where those with more sleep disturbances may be more likely to report low social cohesion and have negative perceptions about their neighborhood environment [49]. The use of both self-reported nSC and sleep dimensions may introduce measurement error. Although we used multiple sleep dimensions, the use of self-reported sleep measures tends to overestimate sleep duration compared to objective measures [50], and the degree as well as direction of measurement error tends to differ depending on the question asked [51]. Additionally, this study did not account for residential history, and changes in the neighborhood environment and perceptions of nSC may impact sleep health. Given the cross-sectional design of the study, it is suggested that future, longitudinal studies should account for the cumulative effect of living in one place, moving neighborhoods, and changes in the neighborhood environment. Another limitation includes unmeasured confounders, especially since sleep disorders, such as sleep apnea, may confound the relationship examined in our study; however, these data were not collected. Finally, the NHIS used a binary (e.g., man/woman) as opposed to a non-binary (man/woman/transgender) definition of sex/gender. 

Despite these limitations, our study has strengths. For instance, we expanded upon the prior literature by investigating multiple sleep dimensions beyond duration as well as the potential modification of the nSC–sleep relationship by age, sex/gender, and race/ethnicity. Another strength of this study includes the use of a nationally representative sample, which enhances the generalizability of our results to the U.S. population of NH-White, NH-Black, Hispanic/Latinx, and Asian adults. The use of the most recent available data collected over multiple years decreased the potential influence attributable to single-year collection periods and increased the sample size. The large sample size allowed robust stratification by three variables both separately and together: age, sex/gender, and race/ethnicity. Another strength of the data includes the NHIS’s quality control procedures, which increase the validity of these findings. Further, the use of a perceived measure to capture nSC is important, because perceived measures are more reflective of the impact that neighborhoods may have on health [52]. Within-neighborhood variations in perceptions of nSC are likely as important as individual-level characteristics; for example, dispositional affects can influence an individual’s perception of their neighborhood’s level of cohesion. Therefore, perceptions of nSC could differentially impact the sleep outcomes of individuals residing in the same neighborhood, and future research, such as assessing how nSC scores cluster among neighbors, is warranted to better understand the implications of this work. Finally, the use of the nonparametric statistic for sleep dimensions can serve as an example of how to model the natural logarithm of average hours of sleep duration and other sleep dimensions. The estimation of prevalence ratios rather than odds ratios is important when outcomes are not rare to avoid the overestimation of prevalence.

## 5. Conclusions

The current research adds knowledge regarding the important role that nSC may have on sleep health. nSC may serve as a key, modifiable neighborhood factor in health promotion programs that are focused on improving sleep. Social-cohesion oriented interventions may potentially mitigate the effect of stress on sleep by enhancing safety, trust, and social support. Research suggests that interventions that improve perceived nSC and other aspects of the social environment may result in improvements in older adults’ and women’s sleep health. Investments in improving the social and cultural qualities of local environments may not benefit all population subgroups uniformly, because we observed that those living in a neighborhood with lower cohesion experienced shorter sleep duration and more sleep disturbances. Future investigation of pathways linking neighborhood factors and sleep is warranted. Specifically, studies may benefit from including other variables of the social neighborhood environment, particularly those related to social resources (e.g., frequency of speaking with others), in order to determine their influence on sleep [53]. Future studies will likely benefit from also examining physical environment variables (e.g., noise, housing density, green space) in relation to sleep, as there may be competing pathways that have different effects on sleep [53]. Future studies should employ an intersectional perspective (or multiple intersecting identities) with the neighborhood environment to understand its influence on health. This approach can help disentangle the complex ways that identities intersect with the neighborhood environment to create social inequality in health. In conclusion, our findings suggest that the social neighborhood environment is associated with sleep duration and disturbances. Our findings underscore the importance of this upstream determinant of sleep health disparities and that the neighborhood environment may be a point of intervention for improving sleep health.

## Figures and Tables

**Table 1:** Age-standardized sociodemographic, health behavior, and clinical characteristics between low, medium, and high neighborhood social cohesion, taken from the National Health Interview Survey, 2013–2018 (n = 167,153) ^a^.

	Neighborhood Social Cohesion			
	Low n = 53,364 (32%)	Medium n = 55,163 (33%)	High n = 58,626 (35%)	Overall n = 167,153 (100%)
**Sociodemographic**				
Age, mean (S.D.), years	44.0 (0.13)	47.1 (0.13)	50.7 (0.14)	47.4 (0.10)
18–30	18.7%	16.2%	13.7%	16.3%
31–50	21.0%	23.5%	26.0%	23.3%
≥50	60.3%	60.3%	60.3%	60.3%
Sex/gender				
Women	52.2%	49.5%	53.0%	51.9%
Race/ethnicity				
NH-White	61.7%	68.1%	77.0%	69.2%
NH-Black	14.1%	11.7%	7.9%	11.1%
Hispanic/Latinx	19.0%	13.9%	10.2%	14.2%
Asian	5.2%	6.3%	5.0%	5.5%
Educational attainment				
<High school	13.6%	9.4%	8.2%	10.3%
High school graduate	29.8%	26.5%	26.1%	27.4%
Some college	31.1%	29.7%	29.7%	30.2%
≥College	25.5%	34.4%	36.0%	32.2%
Annual household income				
<$35,000	37.3%	26.4%	22.8%	28.6%
$35–$74,999	32.1%	30.0%	28.0%	30.1%
≥$75,000	30.6%	43.5%	49.2%	41.3%
Unemployed/not in labor force	43.6%	39.8%	39.6%	40.9%
Occupation class				
Professional/management	17.6%	22.8%	23.6%	21.5%
Support services	44.1%	44.4%	46.5%	45.2%
Laborers	38.3%	32.8%	29.9%	33.4%
Marital status				
Married/living with partner/co-habited	55.3%	62.3%	66.7%	61.7%
Divorced/widowed	24.0%	19.8%	18.4%	20.4%
Single/no live-in partner	20.7%	17.9%	14.9%	17.9%
Region of residence				
Northeast	18.0%	18.9%	18.0%	18.2%
Midwest	21.2%	22.6%	23.7%	22.5%
South	36.3%	35.6%	37.9%	36.7%
West	24.5%	22.9%	20.4%	22.6%
**Health Behaviors**	**Low**	**Medium**	**High**	**Overall**
Sleep duration				
<6 h (very short)	11.8%	7.9%	7.5%	8.9%
<7 h (short)	36.2%	30.5%	27.9%	31.3%
7–9 h (recommended)	59.3%	65.9%	68.2%	64.7%
>9 h (long)	4.5%	3.6%	3.9%	4.0%
Trouble falling asleep (≥3 times/week)	25.4%	18.6%	16.6%	19.9%
Trouble staying asleep (≥3 times/week)	32.2%	26.3%	24.4%	27.4%
Insomnia symptoms ^b^	39.0%	31.6%	29.3%	33.0%
Woke up feeling unrested (≥3 days/week)	50.5%	42.1%	36.9%	42.8%
Sleep medication (≥3 times/week)	11.5%	9.0%	9.1%	9.8%
Smoking status				
Never/quit > 12 months prior	80.4%	84.7%	85.7%	83.7%
Former	1.5%	1.3%	1.2%	1.3%
Current	18.1%	14.0%	13.2%	15.0%
Alcohol status				
Never	20.3%	18.6%	19.3%	19.4%
Former	17.5%	14.6%	14.0%	15.1%
Current	62.2%	66.9%	66.7%	65.5%
Leisure-time physical activity				
Never/unable	37.9%	30.5%	28.5%	32.0%
Does not meet PA guidelines	19.4%	19.1%	18.2%	18.9%
Meets PA guidelines ^c^	42.7%	50.4%	53.3%	49.1%
**Clinical Characteristics**	**Low**	**Medium**	**High**	**Overall**
Health status				
Excellent/very good	49.8%	59.6%	65.4%	58.7%
Good	30.5%	27.6%	23.9%	27.2%
Fair/poor	19.7%	12.8%	10.8%	14.0%
Mental illness ^d^	5.7%	2.6%	2.2%	3.4%
Body Mass Index (BMI)				
Recommended (18.5–< 25 km/m^2^)	30.9%	33.1%	34.8%	33.1%
Overweight (25–29.9 km/m^2^)	34.6%	36.6%	36.5%	36.0%
Obese (≥30 kg/m^2^)	34.5%	30.3%	28.7%	30.9%
Dyslipidemia ^e^	51.0%	48.5%	50.1%	49.7%
Hypertension ^f^	38.6%	35.3%	33.8%	35.7%
Prediabetes/diabetes ^g^	20.9%	17.3%	15.4%	17.6%
“Ideal” cardiovascular health ^h^	7.5%	10.2%	11.5%	9.8%

^a^ Note all estimates are weighted for the survey’s complex sampling design. All estimates are age-standardized to the U.S. 2010 population, except for age. Percentages may not sum to 100 due to missing values or rounding. SE = standard error. ^b^ Insomnia symptoms defined as either trouble falling asleep and/or difficulty maintaining sleep 3+ times a week. ^c^ Meets PA (physical activity) guidelines defined as ≥150 min/week of moderate intensity or ≥75 min/week of vigorous intensity or ≥150 min/week of moderate and vigorous intensity. ^d^ Kessler 6-psychological distress scale score ≥13. ^e^ Dyslipidemia defined as high cholesterol in the 12 months prior to interview. Available for survey years 2011–2017. ^f^ Hypertension defined as respondents ever being told by a doctor that they had hypertension. ^g^ Prediabetes/diabetes defined as respondents ever being told by a doctor that they had diabetes or prediabetic condition. ^h^ “Ideal” cardiovascular health includes never smoking/quit >12 months prior to interview, BMI 18.5–<25 kg/m^2^, meeting physical activity guidelines, and no prior diagnosis of dyslipidemia, hypertension, or diabetes/prediabetes.

**Table 2:** Adjusted prevalence ratios of sleep health by low and medium compared to high neighborhood social cohesion among U.S. racial/ethnic adults by age and sex/gender, National Health Interview Survey, 2013–2018 (n = 167,153).

Sleep Health Dimensions								
**Low Neighborhood Social Cohesion**	Very short <6 vs. 7–9 h	Short <7 vs. 7–9 h	Long >9 vs. 7–9 h	Trouble falling asleep (≥3 times/week)	Trouble staying asleep (≥3 times/week)	Insomnia symptoms ^a^	Woke up feeling unrested (≥3 days/week)	Sleep medication (≥3 times/week)
Women								
NH-White 18–30	1.15 (0.91, 1.46)	1.07 (0.95, 1.20)	1.02 (0.70, 1.50)	**1.21** **(1.07, 1.38)**	**1.3** **(1.15, 1.49)**	**1.23** **(1.11, 1.36)**	**1.26** **(1.16, 1.36)**	**1.31** **(1.01, 1.71)**
NH-White 31–49	**1.39** **(1.21, 1.61)**	**1.19** **(1.11, 1.28)**	1.24 (0.89, 1.73)	**1.19** **(1.09, 1.30)**	**1.26** **(1.17, 1.35)**	**1.21** **(1.14, 1.29)**	**1.16** **(1.11, 1.21)**	1.03 (0.89, 1.20)
NH-White ≥50	**1.40** **(1.25, 1.56)**	**1.28** **(1.21, 1.35)**	1.04 (0.88, 1.23)	**1.35** **(1.27, 1.43)**	**1.22** **(1.17, 1.28)**	**1.23** **(1.18, 1.28)**	**1.30** **(1.25, 1.36)**	1.08 (0.99, 1.18)
NH-Black 18–30	**1.79** **(1.15, 2.79)**	**1.29** **(1.06, 1.58)**	0.64 (0.34, 1.19)	1.29 (0.95, 1.75)	0.94 (0.70, 1.26)	1.22 (0.96, 1.56)	**1.23** **(1.03, 1.46)**	0.90 (0.40, 2.03)
NH-Black 31–49	**1.37** **(1.03, 1.83)**	**1.16** **(1.01, 1.33)**	1.37 (0.71, 2.63)	1.18 (0.95, 1.45)	**1.21** **(1.00, 1.47)**	1.18 (0.99, 1.40)	**1.28** **(1.14, 1.43)**	1.01 (0.70, 1.46)
NH-Black ≥50	1.01 (0.83, 1.24)	1.00 (0.89, 1.11)	1.16 (0.84, 1.60)	**1.26** **(1.06, 1.48)**	**1.17** **(1.02, 1.34)**	**1.20** **(1.07, 1.36)**	**1.18** **(1.06, 1.33)**	1.26 (0.98, 1.63)
Latinx 18–30	1.36 (0.84, 2.19)	**1.31** **(1.02, 1.69)**	0.89 (0.48, 1.64)	**1.42** **(1.03, 1.94)**	1.34 (0.99, 1.83)	**1.37** **(1.06, 1.78)**	**1.24** **(1.08, 1.43)**	**5.51** **(2.02, 15.03)**
Latinx 31–49	**1.53** **(1.13, 2.06)**	**1.34** **(1.16, 1.55)**	1.05 (0.54, 2.05)	**1.25** **(1.04, 1.50)**	**1.31** **(1.08, 1.60)**	**1.26** **(1.07, 1.47)**	**1.30** **(1.17, 1.44)**	0.95 (0.64, 1.42)
Latinx ≥ 50	**1.53** **(1.19, 1.97)**	**1.35** **(1.16, 1.57)**	0.97 (0.60, 1.55)	**1.29** **(1.07, 1.56)**	**1.24** **(1.05, 1.47)**	**1.26** **(1.08, 1.45)**	**1.40** **(1.24, 1.59)**	1.28 (0.95, 1.74)
Asian 18–30	1.57 (0.57, 4.31)	1.24 (0.84, 1.84)	NE	NE	NE	NE	**1.37** **(1.04, 1.81)**	NE
Asian 31–49	1.60 (0.94, 2.72)	**1.29** **(1.02, 1.62)**	NE	**1.60** **(1.03, 2.48)**	**2.20** **(1.45, 3.34)**	**2.09** **(1.49, 2.94)**	**1.25** **(1.04, 1.51)**	0.92 (0.39, 2.19)
Asian ≥50	**1.81** **(1.15, 2.85)**	1.17 (0.96, 1.44)	1.09 (0.49, 2.41)	1.00 (0.71, 1.42)	1.00 (0.75, 1.31)	1.04 (0.81, 1.32)	**1.40** **(1.12, 1.74)**	1.16 (0.57, 2.37)
Men								
NH-White 18–30	1.02 (0.80, 1.30)	1.11 (0.99, 1.26)	1.11 (0.65, 1.91)	**1.39** **(1.16, 1.65)**	**1.50** **(1.22, 1.83)**	**1.42** **(1.22, 1.64)**	**1.24** **(1.12, 1.36)**	**1.68** **(1.15, 2.44)**
NH-White 31–49	1.16 (0.99, 1.36)	**1.16** **(1.08, 1.25)**	1.19 (0.72, 1.95)	**1.24** **(1.11, 1.39)**	**1.16** **(1.05, 1.28)**	**1.22** **(1.13, 1.33)**	**1.27** **(1.20, 1.34)**	1.09 (0.90, 1.34)
NH-White ≥50	**1.48** **(1.28, 1.70)**	**1.18** **(1.10, 1.26)**	0.82 (0.69, 0.97)	**1.29** **(1.18, 1.40)**	**1.27** **(1.20, 1.35)**	**1.26** **(1.20, 1.34)**	**1.40** **(1.33, 1.48)**	**1.12** **(1.00, 1.26)**
NH-Black 18–30	1.70 (0.99, 2.91)	**1.61** **(1.19, 2.17)**	2.14 (0.80, 5.77)	**3.77** **(1.97, 7.22)**	**3.16** **(1.83, 5.47)**	**2.61** **(1.59, 4.31)**	**1.48** **(1.08, 2.02)**	1.17 (0.36, 3.83)
NH-Black 31–49	0.81 (0.58, 1.12)	1.05 (0.90, 1.22)	0.56 (0.27, 1.17)	1.40 (0.99, 1.98)	1.11 (0.85, 1.46)	1.19 (0.93, 1.53)	**1.32** **(1.11, 1.57)**	1.33 (0.67, 2.64)
NH-Black ≥50	1.00 (0.78, 1.28)	1.08 (0.94, 1.23)	1.08 (0.76, 1.54)	**1.37** **(1.08, 1.72)**	1.14 (0.94, 1.37)	**1.18** **(1.00, 1.40)**	**1.30** **(1.11, 1.51)**	1.31 (0.92, 1.85)
Latinx 18–30	1.23 (0.69, 2.19)	1.13 (0.87, 1.46)	1.73 (0.79, 3.78)	1.45 (0.99, 2.13)	1.54 (0.98, 2.44)	**1.49** **(1.06, 2.09)**	**1.26** **(1.05, 1.51)**	1.74 (0.54, 5.60)
Latinx 31–49	1.04 (0.74, 1.45)	1.13 (0.97, 1.32)	0.94 (0.49, 1.79)	**1.40** **(1.07, 1.84)**	1.23 (0.95, 1.60)	**1.41** **(1.13, 1.74)**	**1.19** **(1.05, 1.34)**	**0.57** **(0.34, 0.96)**
Latinx ≥50	1.06 (0.76, 1.47)	1.07 (0.91, 1.27)	0.82 (0.50, 1.36)	1.22 (0.98, 1.50)	1.07 (0.86, 1.34)	**1.21** **(1.01, 1.45)**	**1.22** **(1.05, 1.42)**	1.24 (0.86, 1.79)
Asian 18–30	NE	1.14 (0.77, 1.67)	NE	1.37 (0.70, 2.66)	NE	1.45 (0.77, 2.71)	**1.47** **(1.07, 2.01)**	NE
Asian 31–49	0.57 (0.30, 1.11)	1.05 (0.81, 1.37)	NE	1.19 (0.68, 2.08)	1.43 (0.85, 2.39)	1.33 (0.85, 2.07)	**1.40** **(1.11, 1.76)**	NE
Asian ≥50	1.17 (0.72, 1.91)	**1.31** **(1.04, 1.66)**	NE	1.57 (0.94, 2.61)	**1.74** **(1.16, 2.63)**	1.51 (1.05, 2.17)	1.42 (0.99, 1.63)	1.08 (0.52, 2.24)
**Medium Neighborhood Social Cohesion**	Very short <6 vs. 7–9 h	Short <7 vs. 7–9 h	Long >9 vs. 7–9 h	Trouble falling asleep (≥3 times/week)	Trouble staying asleep (≥3 times/week)	Insomnia symptoms ^a^	Woke up feeling unrested (≥3 days/week)	Sleep medication (≥3 times/week)
Women								
NH-White 18–30	0.95 (0.73, 1.24)	1.01 (0.90, 1.14)	0.81 (0.52, 1.25)	1.11 (0.98, 1.27)	**1.18** **(1.03, 1.35)**	**1.17** **(1.05, 1.30)**	**1.16** **(1.07, 1.25)**	0.77 (0.57, 1.04)
NH-White 31–49	1.03 (0.87, 1.22)	1.05 (0.97, 1.13)	0.91 (0.64, 1.31)	1.06 (0.97, 1.15)	**1.17** **(1.09, 1.26)**	**1.14** **(1.07, 1.22)**	**1.08** **(1.03, 1.13)**	0.96 (0.83, 1.12)
NH-White ≥50	0.99 (0.87, 1.11)	**1.11** **(1.05, 1.18)**	**0.86** **(0.74, 0.99)**	**1.14** **(1.07, 1.21)**	**1.08** **(1.03, 1.13)**	**1.08** **(1.04, 1.12)**	**1.12** **(1.07, 1.17)**	0.97 (0.90, 1.05)
NH-Black 18–30	1.54 (0.89, 2.64)	**1.27** **(1.00, 1.60)**	0.90 (0.48, 1.70)	1.15 (0.79, 1.67)	0.88 (0.62, 1.26)	1.10 (0.81, 1.49)	1.07 (0.88, 1.31)	0.76 (0.31, 1.87)
NH-Black 31–49	1.06 (0.78, 1.44)	1.02 (0.89, 1.18)	1.25 (0.62, 2.51)	0.94 (0.73, 1.20)	0.97 (0.79, 1.19)	0.96 (0.80, 1.16)	**1.15** **(1.01, 1.30)**	0.88 (0.59, 1.31)
NH-Black ≥50	0.80 (0.64, 1.01)	**0.90** **(0.80, 1.00)**	1.14 (0.86, 1.53)	1.11 (0.94, 1.30)	1.06 (0.92, 1.21)	1.07 (0.94, 1.20)	1.07 (0.96, 1.19)	1.27 (0.98, 1.63)
Latinx 18–30	1.04 (0.62, 1.76)	1.14 (0.86, 1.51)	0.81 (0.41, 1.61)	0.95 (0.68, 1.35)	0.95 (0.67, 1.34)	1.01 (0.76, 1.34)	1.07 (0.91, 1.26)	**2.94** **(1.03, 8.40)**
Latinx 31–49	1.15 (0.81, 1.63)	1.16 (0.99, 1.36)	0.75 (0.37, 1.50)	1.08 (0.88, 1.33)	1.15 (0.93, 1.42)	1.10 (0.93, 1.31)	**1.19** **(1.06, 1.33)**	0.66 (0.43, 1.01)
Latinx ≥50	1.05 (0.79, 1.40)	1.12 (0.95, 1.32)	0.69 (0.38, 1.23)	1.12 (0.91, 1.38)	1.08 (0.90, 1.31)	1.11 (0.94, 1.31)	**1.34** **(1.18, 1.54)**	1.26 (0.93, 1.70)
Asian 18–30	1.44 (0.53, 3.94)	0.75 (0.48, 1.17)	NE	NE	NE	NE	1.03 (0.78, 1.37)	NE
Asian 31–49	0.65 (0.36, 1.16)	1.04 (0.81, 1.33)	NE	1.24 (0.81, 1.91)	1.27 (0.82, 1.95)	1.27 (0.90, 1.78)	1.02 (0.85, 1.21)	0.46 (0.21, 1.01)
Asian ≥50	1.12 (0.71, 1.78)	0.89 (0.73, 1.10)	0.70 (0.34, 1.42)	0.86 (0.60, 1.22)	0.77 (0.56, 1.07)	0.78 (0.60, 1.02)	1.03 (0.83, 1.28)	1.22 (0.66, 2.25)
Men								
NH-White 18–30	**0.75** **(0.56, 1.00)**	1.04 (0.92, 1.17)	1.47 (0.89, 2.41)	1.07 (0.88, 1.31)	1.22 (0.97, 1.54)	1.10 (0.92, 1.30)	**1.11** **(1.00, 1.23)**	1.37 (0.90, 2.09)
NH-White 31–49	1.15 (0.98, 1.34)	**1.07** **(1.00, 1.14)**	1.50 (0.93, 2.41)	**1.20** **(1.07, 1.35)**	1.10 (0.99, 1.21)	**1.11** **(1.02, 1.20)**	**1.14** **(1.08, 1.21)**	**1.34** **(1.11, 1.61)**
NH-White ≥50	**1.18** **(1.03, 1.35)**	**1.07** **(1.00, 1.14)**	0.90 (0.77, 1.05)	**1.11** **(1.01, 1.21)**	**1.12** **(1.06, 1.19)**	**1.11** **(1.06, 1.17)**	**1.24** **(1.18, 1.30)**	1.04 (0.93, 1.16)
NH-Black 18–30	1.28 (0.71, 2.31)	1.28 (0.93, 1.77)	1.49 (0.42, 5.34)	**2.82** **(1.43, 5.58)**	**2.35** **(1.28, 4.31)**	**2.04** **(1.19, 3.50)**	1.29 (0.94, 1.77)	0.40 (0.08, 1.94)
NH-Black 31–49	0.94 (0.68, 1.31)	1.03 (0.89, 1.20)	**0.36** **(0.16, 0.78)**	1.15 (0.81, 1.64)	0.84 (0.64, 1.10)	0.92 (0.72, 1.19)	**1.23** **(1.04, 1.46)**	0.99 (0.50, 1.97)
NH-Black ≥50	0.83 (0.63, 1.09)	1.03 (0.90, 1.17)	0.80 (0.57, 1.13)	**1.39** **(1.10, 1.77)**	**1.19** **(1.00, 1.42)**	**1.19** **(1.01, 1.40)**	**1.22** **(1.05, 1.41)**	0.89 (0.61, 1.31)
Latinx 18–30	1.22 (0.68, 2.22)	1.09 (0.83, 1.42)	1.39 (0.61, 3.19)	1.20 (0.81, 1.79)	1.18 (0.74, 1.88)	1.27 (0.90, 1.80)	1.10 (0.91, 1.33)	2.55 (0.96, 6.76)
Latinx 31–49	0.91 (0.64, 1.28)	1.08 (0.92, 1.26)	**0.38** **(0.18, 0.82)**	1.07 (0.80, 1.43)	0.85 (0.63, 1.15)	1.02 (0.80, 1.31)	1.03 (0.89, 1.18)	**0.32** **(0.17, 0.60)**
Latinx ≥50	0.77 (0.55, 1.09)	0.91 (0.76, 1.08)	0.76 (0.47, 1.21)	0.83 (0.64, 1.07)	0.94 (0.75, 1.17)	0.94 (0.76, 1.15)	1.01 (0.85, 1.20)	0.88 (0.60, 1.29)
Asian 18–30	NE	0.73 (0.48, 1.11)	NE	0.73 (0.35, 1.52)	NE	0.68 (0.33, 1.38)	1.13 (0.81, 1.58)	NE
Asian 31–49	0.84 (0.44, 1.61)	0.92 (0.72, 1.17)	NE	1.16 (0.67, 2.02)	0.78 (0.49, 1.26)	1.00 (0.66, 1.52)	1.29 (1.04, 1.61)	NE
Asian ≥50	1.28 (0.77, 2.13)	1.31 (1.05, 1.62)	NE	1.22 (0.73, 2.04)	**1.64** **(1.08, 2.49)**	1.34 (0.92, 1.96)	1.27 (0.99, 1.63)	1.15 (0.56, 2.33)

NE = not estimable. Model adjusted for educational attainment (<high school, high school graduate, some college, ≥college), annual household income (<$35,000, $35,000–$74,999, $75,000+), occupational class (professional/management, support services, laborers), region of residence (Northeast, Midwest, South, West), alcohol consumption (never, former, current), serious mental illness (Kessler-6 psychological distress scale score ≥13), “ideal” cardiovascular health (never smoking/quit >12 months prior to interview, BMI 18.5 to <25 kg/m^2^, meeting physical activity guidelines, and no prior diagnosis of dyslipidemia, hypertension, or diabetes/prediabetes), marital/co-habiting status (married/living with partner or cohabitating, divorced/widowed/separated, single/no live-in partner), employment status (unemployed, employed), and health status (excellent/very good, good, fair/poor). Reference level: high neighborhood cohesion. Note. All estimates are weighted for the survey’s complex sampling design. Boldface indicates statistically significant results at the 0.05 level. Insomnia symptoms defined as either trouble falling asleep and/or difficulty maintaining sleep 3+ times a week.

## Supplementary Materials

The following are available online at https://www.mdpi.com/1660-4601/17/24/9475/s1, Figure S1: Flow Chart of Participant Selection, Table S1: Age-standardized Sociodemographic, Health Behavior, and Clinical Characteristics between Low, Medium, and High Neighborhood Social Cohesion Among 18–30, 31–49, and ≥50 years old, National Health Interview Survey, 2013–2018 (N = 167,153), Table S2: Age-standardized Sociodemographic, Health Behavior, and Clinical Characteristics between Low, Medium, and High Neighborhood Social Cohesion Among Women and Men, National Health Interview Survey, 2013–2018, Table S3: Age-standardized Sociodemographic, Health Behavior, and Clinical Characteristics between Low, Medium, and High Neighborhood Social Cohesion Among Racial/Ethnic Groups, National Health Interview Survey, 2013–2018, Table S4: Age-standardized Sociodemographic between Neighborhood Social Cohesion Scale, National Health Interview Survey, 2013–2018 (N = 167,153).

## References

[1] Colten H.R., Altevogt B.M. (2006). Institute of Medicine Report. Sleep Disorders and Sleep Deprivation: An Unmet Public Health Problem.

[2] Liu Y. (2016). Prevalence of healthy sleep duration among adults US 2014. Morb. Mortal. Wkly. Rep..

[3] Kaufmann C.N., Canham S.L., Mojtabai R., Gum A.M., Dautovich N.D., Kohn R., Spira A.P. (2013). Insomnia and health services utilization in middle-aged and older adults: Results from the Health and Retirement Study. J. Gerontol. A Biol. Sci. Med. Sci..

[4] Theorell-Haglow J., Miller C.B., Bartlett D.J., Yee B.J., Openshaw H.D., Grunstein R.R. (2018). Gender differences in obstructive sleep apnoea, insomnia and restless legs syndrome in adults–What do we know? A clinical update. Sleep Med. Rev..

[5] Chen X., Wang R., Zee P., Lutsey P.L., Javaheri S., Alcantara C., Jackson C.L., Williams M.A., Redline S. (2015). Racial/Ethnic Differences in Sleep Disturbances: The Multi-Ethnic Study of Atherosclerosis (MESA). Sleep.

[6] Jackson C.L., Redline S., Emmons K.M. (2015). Sleep as a potential fundamental contributor to disparities in cardiovascular health. Annu. Rev. Public Health.

[7] Carnethon M.R., De Chavez P.J., Zee P.C., Kim K.Y., Liu K., Goldberger J.J., Ng J., Knutson K.L. (2016). Disparities in sleep characteristics by race/ethnicity in a population-based sample: Chicago Area Sleep Study. Sleep Med..

[8] Whinnery J., Jackson N., Rattanaumpawan P., Grandner M.A. (2014). Short and long sleep duration associated with race/ethnicity, sociodemographics, and socioeconomic position. Sleep.

[9] Garbarino S., Magnavita N. (2019). Sleep problems are a strong predictor of stress-related metabolic changes in police officers. A prospective study. PLoS ONE.

[10] Desantis A.S., Diez Roux A.V., Moore K., Baron K.G., Mujahid M.S., Nieto F.J. (2013). Associations of neighborhood characteristics with sleep timing and quality: The Multi-Ethnic Study Of Atherosclerosis. Sleep.

[11] Johnson D.A., Brown D.L., Morgenstern L.B., Meurer W.J., Lisabeth L.D. (2015). The association of neighborhood characteristics with sleep duration and daytime sleepiness. Sleep Health.

[12] Kawachi I., Berkman L.F., Kawachi I., Berkman L.F. (2000). Social Cohesion, Social Capital, and Health. Social Epidemiology.

[13] Hale L., Hill T.D., Friedman E., Nieto F.J., Galvao L.W., Engelman C.D., Malecki K.M., Peppard P.E. (2013). Perceived neighborhood quality, sleep quality, and health status: Evidence from the Survey of the Health of Wisconsin. Soc. Sci. Med..

[14] Johnson D.A., Al-Ajlouni Y.A., Duncan S.D.T. (2019). Connecting Neighborhoods and Sleep Health. Soc. Epidemiol. Sleep.

[15] Hirotsu C., Tufik S., Andersen M.L. (2015). Interactions between sleep, stress, and metabolism: From physiological to pathological conditions. Sleep Sci..

[16] Magnavita N., Di Stasio E., Capitanelli I., Lops E.A., Chirico F., Garbarino S. (2019). Sleep Problems and Workplace Violence: A Systematic Review and Meta-Analysis. Front. Neurosci..

[17] Johnson D.A., Simonelli G., Moore K., Billings M., Mujahid M.S., Rueschman M., Kawachi I., Redline S., Diez Roux A.V., Patel S.R. (2017). The Neighborhood Social Environment and Objective Measures of Sleep in the Multi-Ethnic Study of Atherosclerosis. Sleep.

[18] Lang I.A., Llewellyn D.J., Langa K.M., Wallace R.B., Huppert F.A., Melzer D. (2008). Neighborhood deprivation, individual socioeconomic status, and cognitive function in older people: Analyses from the English Longitudinal Study of Ageing. J. Am. Geriatr. Soc..

[19] Adams M.A., Sallis J.F., Conway T.L., Frank L.D., Saelens B.E., Kerr J., Cain K.L., King A.C. (2012). Neighborhood environment profiles for physical activity among older adults. Am. J. Health Behav..

[20] Chen-Edinboro L.P., Kaufmann C.N., Augustinavicius J.L., Mojtabai R., Parisi J.M., Wennberg A.M., Smith M.T., Spira A.P. (2014). Neighborhood physical disorder, social cohesion, and insomnia: Results from participants over age 50 in the Health and Retirement Study. Int. Psychogeriatr..

[21] Guilcher S.J.T., Kaufman-Shriqui V., Hwang J., O’Campo P., Matheson F.I., Glazier R.H., Booth G.L. (2017). The association between social cohesion in the neighborhood and body mass index (BMI): An examination of gendered differences among urban-dwelling Canadians. Prev. Med..

[22] Molinari C., Ahern M., Hendryx M. (1998). Relationship of Community Quality to Health of Women and Men. Soc. Sci. Med..

[23] Almeida J., Kawachi I., Molnar B.E., Subramanian S.V. (2009). A multilevel analysis of social ties and social cohesion among Latinos and their neighborhoods: Results from Chicago. J. Urban Health.

[24] Hobson-Prater T., Leech T.G.J. (2012). The Significance of Race for Neighborhood Social Cohesion: Perceived Difficulty of Collective Action in Majority Black Neighborhoods. J. Soc. Soc. Welfare.

[25] LaVeist T.A. (2000). On the study of race, racism and health: A shift from description to explanation. Int. J. Health Serv..

[26] House J.S. (2002). Understanding social factors and inequalities in health: 20th century progress and 21st century prospects. J. Health Soc. Behav..

[27] Johnson D.A., Jackson C.L., Williams N.J., Alcantara C. (2019). Are sleep patterns influenced by race/ethnicity—A marker of relative advantage or disadvantage? Evidence to date. Nat. Sci. Sleep.

[28] Slopen N., Williams D.R. (2014). Discrimination, other psychosocial stressors, and self-reported sleep duration and difficulties. Sleep.

[29] IPUMS Health Surveys: National Health Interview Survey, Version 6.2. https://nhis.ipums.org/nhis/.

[30] (2016). National Health Interview Survey Description.

[31] Sampson R.J., Raudenbush S., Earls F. (1997). Neighborhoods and violent crime: A multielvel study of collective efficacy. Science.

[32] Young M.C., Gerber M.W., Ash T., Horan C.M., Taveras E.M. (2018). Neighborhood social cohesion and sleep outcomes in the Native Hawaiian and Pacific Islander National Health Interview Survey. Sleep.

[33] Hirshkowitz M., Whiton K., Albert S.M., Alessi C., Bruni O., DonCarlos L., Hazen N., Herman J., Adams Hillard P.J., Katz E.S. (2015). National Sleep Foundation’s updated sleep duration recommendations: Final report. Sleep Health.

[34] Piercy K.L., Troiano R.P., Ballard R.M., Carlson S.A., Fulton J.E., Galuska D.A., George S.M., Olson R.D. (2018). The Physical Activity Guidelines for Americans. JAMA.

[35] Kessler R.C., Barker P.R., Colpe L.J., Epstein J.F., Gfroerer J.C., Hiripe E., Howes M.J., Normand S.T., Manderscheid R.W., Waltersm E.E. (2003). Screenings for Serious Mental Illness in the General Population. Arch. Gen. Psychiatry.

[36] Lloyd-Jones D.M., Hong Y., Labarthe D., Mozaffarian D., Appel L.J., Van Horn L., Greenlund K., Daniels S., Nichol G., Tomaselli G.F. (2010). Defining and setting national goals for cardiovascular health promotion and disease reduction: The American Heart Association’s strategic Impact Goal through 2020 and beyond. Circulation.

[37] Barros A.J., Hirakata V.N. (2003). Alternatives for logistic regression in cross-sectional studies: An empirical comparison of models that directly estimate the prevalence ratio. BMC Med. Res. Methodol..

[38] Bassett E., Moore S. (2014). Neighbourhood disadvantage, network capital and restless sleep: Is the association moderated by gender in urban-dwelling adults?. Soc. Sci. Med..

[39] Kavanagh A.M., Bentley R., Turrell G., Broom D.H., Subramanian S.V. (2006). Does gender modify associations between self rated health and the social and economic characteristics of local environments?. J. Epidemiol. Community Health.

[40] Stafford M.A.I., McMunn A., De Vogli R. (2011). Neighbourhood social environment and depressive symptoms in mid-life and beyond. Ageing Soc..

[41] Simonelli G., Patel S.R., Rodriguez-Espinola S., Perez-Chada D., Salvia A., Cardinali D.P., Vigo D.E. (2015). The impact of home safety on sleep in a Latin American country. Sleep Health.

[42] Xiao Q., Hale L. (2018). Neighborhood socioeconomic status, sleep duration, and napping in middle-to-old aged US men and women. Sleep.

[43] Johnson D.A., Lisabeth L., Hickson D., Johnson-Lawrence V., Samdarshi T., Taylor H., Diez Roux A.V. (2016). The Social Patterning of Sleep in African Americans: Associations of Socioeconomic Position and Neighborhood Characteristics with Sleep in the Jackson Heart Study. Sleep.

[44] Murillo R., Ayalew L., Hernandez D.C. (2019). The association between neighborhood social cohesion and sleep duration in Latinos. Ethn. Health.

[45] Troxel W.M., DeSantis A., Richardson A.S., Beckman R., Ghosh-Dastidar B., Nugroho A., Hale L., Buysse D.J., Buman M.P., Dubowitz T. (2018). Neighborhood disadvantage is associated with actigraphy-assessed sleep continuity and short sleep duration. Sleep.

[46] Neergheen V.L., Topel M., Van Dyke M.E., Sullivan S., Pemu P.E., Gibbons G.H., Vaccarino V., Quyyumi A.A., Lewis T.T. (2019). Neighborhood social cohesion is associated with lower levels of interleukin-6 in African American women. Brain Behav. Immun..

[47] Nowakowski S., Matthews K.A., von Kanel R., Hall M.H., Thurston R.C. (2018). Sleep characteristics and inflammatory biomarkers among midlife women. Sleep.

[48] McNeill L.H., Kreuter M.W., Subramanian S.V. (2006). Social environment and physical activity: A review of concepts and evidence. Soc. Sci. Med..

[49] Nam S., Whittemore R., Jung S., Latkin C., Kershaw T., Redeker N.S. (2018). Physical neighborhood and social environment, beliefs about sleep, sleep hygiene behaviors, and sleep quality among African Americans. Sleep Health.

[50] Jackson C.L., Patel S.R., Jackson W.B., Lutsey P.L., Redline S. (2018). Agreement between self-reported and objectively measured sleep duration among white, black, Hispanic, and Chinese adults in the United States: Multi-Ethnic Study of Atherosclerosis. Sleep.

[51] Jackson C.L., Ward J.B., Johnson D.A., Sims M., Wilson J., Redline S. (2020). Concordance between self-reported and actigraphy-assessed sleep duration among African-American adults: Findings from the Jackson Heart Sleep Study. Sleep.

[52] Weden M.M., Carpiano R.M., Robert S.A. (2008). Subjective and objective neighborhood characteristics and adult health. Soc. Sci. Med..

[53] Jackson C.L., Gaston S.A. (2019). The impact of environmental exposures on sleep. Sleep and Health.

