# Supplementary material for: Neighborhood Social Cohesion and Sleep Health by Age, Sex/Gender, and Race/Ethnicity in the United States
Source: Int J Environ Res Public Health. 2020 Dec 17;17(24):9475. doi: 10.3390/ijerph17249475 (PMC7767208; doi:10.3390/ijerph17249475)

Supplemental Table 1. Age-standardized Sociodemographic, Health Behavior, and Clinical Characteristics between Low, Medium, and High Neighborhood Social Cohesion Among 18–30, 31–49, and ≥50 years old, National Health Interview Survey, 2013–2018 (N=167,153) [a.]

	**Neighborhood Social Cohesion**								
	**Low** *n*=53,364 (32%)			**Medium** *n*=55,163 (33%)			**High** *n*=58,626 (35%)		
**Sociodemographic**	18–30 *n*=13,123	31–50 *n*=17,036	≥50 *n*=23,205	18–30 *n*=10,073	31–50 *n*=17,003	≥50 *n*=28,087	18–30 *n*=7,308	31–50 *n*=16,130	≥50 *n*=35,188
Sex/gender									
Women	51.2%	52.4%	54.1%	47.1%	49.1%	50.4%	50.8%	51.4%	54.1%
Race/ethnicity									
NH-White	52.6%	51.6%	68.0%	59.5%	60.3%	73.5%	69.6%	71.7%	81.0%
NH-Black	16.4%	15.9%	12.8%	12.5%	12.5%	11.1%	10.2%	7.5%	7.5%
Hispanic/Latinx	25.5%	26.2%	14.4%	21.5%	18.8%	10.0%	14.8%	14.1%	7.4%
Asian	5.5%	6.3%	4.8%	6.5%	8.4%	5.4%	5.3%	6.7%	4.1%
Educational attainment									
<High school	10.0%	13.3%	14.8%	8.0%	8.7%	10.0%	7.2%	6.8%	9.1%
High school graduate	30.0%	27.1%	30.6%	27.9%	22.0%	27.8%	27.0%	20.5%	28.2%
Some college	38.8%	29.8%	29.1%	38.0%	27.1%	28.5%	41.4%	27.4%	28.1%
≥College	21.1%	29.8%	25.4%	26.1%	42.1%	33.7%	24.4%	45.3%	34.6%
Annual household income									
<$35,000	42.4%	31.7%	37.6%	32.5%	20.6%	27.1%	27.3%	14.6%	25.3%
$35-$74,999	33.6%	32.5%	31.5%	32.4%	28.0%	30.3%	29.1%	24.8%	29.2%
≥$75,000	24.0%	35.8%	30.9%	35.1%	51.4%	42.7%	43.6%	60.6%	45.5%
Unemployed/not in labor force	30.4%	23.2%	54.8%	30.3%	18.1%	50.7%	31.2%	18.0%	50.8%
Occupational class									
Professional/management	13.6%	19.7%	18.0%	16.6%	26.5%	22.9%	14.6%	28.0%	23.5%
Support services	48.6%	43.5%	43.0%	47.2%	42.8%	44.3%	50.2%	44.8%	46.5%
Laborers	37.8%	36.8%	39.0%	36.1%	30.7%	32.7%	35.1%	27.2%	30.0%
Marital/co-habiting status									
Married/living with partner or cohabited	36.4%	65.0%	57.8%	34.7%	72.6%	65.7%	32.8%	78.9%	69.1%
Divorced/widowed	3.4%	15.4%	33.3%	2.6%	12.5%	27.2%	3.1%	10.3%	25.3%
Single/no live-in partner	60.2%	19.6%	8.9%	62.7%	15.0%	7.1%	64.0%	10.8%	5.5%
Region of residence									
Northeast	15.1%	16.3%	19.5%	16.8%	17.4%	20.0%	17.0%	17.4%	18.4%

Midwest	22.2%	20.1%	21.2%	24.0%	22.4%	22.3%	25.2%	23.5%	23.5%
South	36.1%	36.9%	36.2%	35.6%	35.6%	35.6%	37.1%	37.2%	38.3%
West	26.6%	26.7%	23.1%	23.5%	24.6%	22.1%	20.7%	21.9%	19.7%
**Health Behaviors**		**Low**			**Medium**			**High**	
	**18–30**	**31–50**	**≥50**	**18–30**	**31–50**	**≥50**	**18–30**	**31–50**	**≥50**
Sleep duration									
<6 hours (very short)	9.8%	12.1%	12.3%	6.4%	9.0%	7.8%	6.4%	8.1%	7.5%
6–7 hours (short)	21.5%	27.9%	24.1%	19.5%	25.6%	22.4%	17.0%	23.5%	19.8%
7–9 hours (recommended)	64.6%	57.4%	58.4%	70.1%	63.9%	65.5%	72.6%	66.7%	67.8%
>9 hours (long)	4.2%	2.6%	5.3%	4.0%	1.6%	4.3%	3.9%	1.8%	4.8%
Trouble falling asleep (≥3 times/week)	24.4%	24.8%	25.9%	17.8%	18.4%	18.9%	15.5%	16.4%	16.9%
Trouble staying asleep (≥3 times/week)	21.0%	29.5%	36.7%	15.4%	23.4%	30.3%	13.6%	22.0%	27.9%
Insomnia symptoms [b]	31.2%	36.5%	42.4%	23.7%	29.1%	34.7%	20.8%	27.0%	32.2%
Woke up feeling unrested (≥3 days/week)	51.8%	56.5%	48.0%	43.0%	49.6%	39.0%	38.1%	44.7%	33.3%
Sleep medication (≥3 times/week)	5.3%	8.5%	14.4%	3.5%	6.7%	11.3%	3.5%	6.8%	11.5%
Smoking status									
Never/quit >12 months prior	80.2%	77.2%	81.6%	84.4%	82.0%	85.9%	84.9%	83.5%	86.8%
Quit ≤12 months	2.2%	1.7%	1.1%	2.1%	1.8%	0.9%	2.2%	1.4%	0.9%
Current	17.5%	21.1%	17.3%	13.5%	16.2%	13.2%	13.0%	15.1%	12.4%
Alcohol status									
Never	25.9%	17.1%	19.7%	28.3%	15.1%	17.3%	30.4%	15.7%	18.4%
Former	5.5%	11.7%	23.3%	4.9%	10.0%	18.9%	5.4%	9.4%	17.9%
Current	68.6%	71.1%	57.1%	66.8%	75.0%	63.7%	64.1%	74.9%	63.7%
Leisure-time physical activity									
Never/unable	26.4%	33.2%	43.1%	22.6%	25.1%	34.7%	21.3%	22.8%	32.5%
Does not meet PA guidelines	16.7%	18.9%	20.4%	14.8%	19.1%	20.2%	14.3%	17.5%	19.5%
Meets PA guidelines [c]	56.9%	47.9%	36.5%	62.5%	55.8%	45.1%	64.4%	59.6%	48.0%
**Clinical Characteristics**		**Low**			**Medium**			**High**	
	**18–30**	**31–50**	**≥50**	**18–30**	**31–50**	**≥50**	**18–30**	**31–50**	**≥50**
Health status									
Excellent/very good	70.3%	58.2%	40.5%	77.2%	68.2%	51.6%	82.9%	73.4%	57.9%
Good	23.2%	29.3%	33.2%	19.0%	24.2%	31.2%	13.9%	21.0%	27.4%
Fair/poor	6.5%	12.6%	26.3%	3.8%	7.6%	17.2%	3.2%	5.6%	14.7%
Mental illness [d]	4.2%	5.6%	6.2%	2.3%	2.5%	2.7%	2.0%	2.2%	2.3%
Body Mass Index (BMI)									

Recommended BMI (18.5-<25 km/m^2)	45.5%	28.0%	27.4%	48.8%	32.1%	29.3%	50.0%	34.0%	31.8%
Overweight (25–29.9 km/m^2)	28.5%	35.8%	36.1%	29.5%	36.6%	38.5%	28.4%	35.8%	38.7%
Obese (≥30 kg/m^2)	26.0%	36.2%	36.5%	21.7%	31.3%	32.2%	21.6%	30.2%	29.6%
Dyslipidemia [e]	12.2%	39.9%	64.9%	12.1%	36.1%	61.6%	12.9%	37.8%	62.3%
Hypertension [f]	7.3%	21.9%	54.1%	7.3%	18.4%	49.5%	6.1%	17.9%	47.0%
Prediabetes/diabetes [g]	3.6%	11.7%	29.4%	3.3%	9.3%	24.1%	2.2%	8.1%	21.5%
"Ideal" cardiovascular health [h]	18.4%	8.3%	3.9%	23.4%	12.1%	6.0%	25.7%	13.9%	7.2%

[a] Note all estimates are weighted for the survey's complex sampling design. All estimates are age-standardized to the U.S. 2010 population, except for age.Percentage may not sum to 100 due to missing values or rounding. [b] Insomnia symptoms defined as either trouble falling asleep and/or difficulty maintaining sleep 3+ times a week.[c] Meets PA guidelines defined as ≥150 minutes/week of moderate intensity or ≥75 minutes/week of vigorous intensity or ≥150 minutes/week of moderate and vigorous intensity. [d] Kessler 6-psychological distress scale score ≥13.[e] Dyslipidemia defined as high cholesterol in the 12 months prior to interview. Available for survey years 2011–2017. [f] Hypertension defined as ever told by a doctor had hypertension. [g] Prediabetes/diabetes defined as ever told by a doctor had diabetes or prediabetic condition. [h] "Ideal" cardiovascular health includes never smoking/quit >12 months prior to interview, BMI 18.5 - <25 kg/m^2, meeting physical activity guidelines, and no prior diagnosis of dyslipidemia, hypertension, or diabetes/prediabetes. .

Supplemental Table 2. Age-standardized Sociodemographic, Health Behavior, and Clinical Characteristics between Low, Medium, and High Neighborhood Social Cohesion Among Women and Men, National Health Interview Survey, 2013–2018 (N=167,153) [a.]

	Neighborhood Social Cohesion					
	Low *n*=53,364 (32%)		Medium *n*=55,163 (33%)		High *n*=58,626 (35%)	
**Sociodemographic**	Women *n*=29,867	Men *n*=23,497	Women *n*=29,031	Men *n*=26,132	Women *n*=32,790	Men *n*=25,836
Age, mean (S.E.)	44.5 (0.17)	43.4 (0.17)	47.8 (0.16)	46.5 (0.17)	51.4 (0.17)	49.9 (0.17)
18–30 years	18.4%	18.9%	15.8%	16.5%	13.6%	13.8%
31–50 years	21.2%	20.8%	23.9%	23.1%	26.1%	25.8%
≥50 years	60.3%	60.3%	60.3%	60.3%	60.3%	60.3%
Race/ethnicity						
NH-White	60.4%	63.2%	67.7%	68.5%	76.9%	77.1%
NH-Black	15.3%	12.8%	12.5%	10.9%	7.9%	7.9%
Hispanic/Latinx	18.8%	19.1%	13.3%	14.5%	10.0%	10.3%
Asian	5.5%	4.9%	6.4%	6.1%	5.3%	4.7%
Educational attainment						
<High school	13.8%	13.4%	9.2%	9.6%	7.7%	8.9%
High school graduate	29.2%	30.3%	25.8%	27.0%	25.0%	27.0%
Some college	32.1%	30.0%	30.7%	28.7%	31.1%	28.2%
≥College	24.9%	26.3%	34.3%	34.7%	36.3%	35.9%
Annual household income						
<$35,000	40.3%	33.8%	28.6%	24.2%	25.0%	20.2%
$35-$74,999	31.7%	32.5%	30.3%	29.9%	27.4%	28.7%
≥$75,000	27.9%	33.7%	41.2%	45.9%	47.6%	51.0%
Unemployed/not in labor force	47.9%	38.8%	44.7%	35.0%	44.6%	34.1%
Occupational class						
Professional/management	14.8%	20.8%	18.4%	27.1%	18.9%	28.8%
Support services	60.3%	26.3%	63.1%	26.6%	64.3%	26.9%
Laborers	24.9%	52.9%	18.5%	46.3%	16.8%	44.3%
Marital/co-habiting status						
Married/living with partner or co habited	51.7%	59.8%	58.9%	66.0%	64.0%	70.1%
Divorced/widowed	28.9%	18.0%	24.2%	15.1%	22.4%	13.5%
Single/no live-in partner	19.4%	22.2%	16.9%	18.9%	13.5%	16.4%

Region of residence						
Northeast	18.2%	17.8%	19.1%	18.7%	18.3%	17.6%
Midwest	21.2%	21.1%	22.5%	22.7%	23.0%	24.5%
South	37.0%	35.6%	36.1%	35.2%	38.1%	37.6%
West	23.6%	25.6%	22.3%	23.5%	20.5%	20.4%
**Health Behaviors**	**Low**		**Medium**		**High**	
	**Women**	**Men**	**Women**	**Men**	**Women**	**Men**
Sleep duration						
<6 hours (very short)	13.1%	10.4%	7.8%	7.9%	7.7%	7.3%
6–7 hours (short)	24.5%	24.3%	22.6%	22.6%	19.8%	21.0%
7–9 hours (recommended)	57.6%	61.2%	66.0%	65.8%	68.5%	67.9%
>9 hours (long)	4.8%	4.1%	3.6%	3.6%	3.9%	3.9%
Trouble falling asleep (≥3 times/week)	29.9%	20.2%	22.2%	14.9%	20.0%	12.8%
Trouble staying asleep (≥3 times/week)	36.2%	27.8%	29.7%	23.0%	27.7%	20.7%
Insomnia symptoms [b]	43.7%	33.8%	36.1%	27.3%	33.5%	24.6%
Woke up feeling unrested (≥3 days/week)	54.5%	46.1%	45.4%	39.0%	40.5%	33.0%
Sleep medication (≥3 times/week)	13.7%	9.0%	10.6%	7.3%	11.2%	6.9%
Smoking status						
Never/quit >12 months prior	82.7%	77.8%	86.4%	83.1%	87.4%	83.7%
Quit ≤12 months	1.4%	1.6%	1.1%	1.5%	1.0%	1.4%
Current	15.9%	20.6%	12.5%	15.3%	11.5%	15.0%
Alcohol status						
Never	26.0%	13.7%	23.5%	13.6%	23.7%	14.2%
Former	16.8%	18.4%	14.1%	15.0%	13.1%	15.1%
Current	57.2%	67.9%	62.4%	71.3%	63.2%	70.7%
Leisure-time physical activity						
Never/unable	39.6%	35.9%	32.4%	28.5%	29.1%	27.7%
Does not meet PA guidelines	21.1%	17.4%	21.0%	17.3%	20.1%	16.2%
Meets PA guidelines [c]	39.3%	46.7%	46.6%	54.2%	50.8%	56.1%
**Clinical Characteristics**	**Low**		**Medium**		**High**	
	**Women**	**Men**	**Women**	**Men**	**Women**	**Men**
Health status						
Excellent/very good	48.3%	51.4%	59.1%	60.1%	65.4%	65.3%
Good	31.2%	29.7%	28.1%	27.1%	23.8%	24.0%
Fair/poor	20.5%	18.9%	12.8%	12.8%	10.8%	10.7%

Mental illness [d]	6.8%	4.5%	3.1%	2.1%	2.6%	1.8%
Body Mass Index (BMI)						
Recommended BMI (18.5-<25 km/m^2)	34.4%	27.1%	39.8%	26.9%	42.6%	26.6%
Overweight (25–29.9 km/m^2)	30.0%	39.6%	30.1%	42.7%	30.2%	43.3%
Obese (≥30 kg/m^2)	35.7%	33.3%	30.1%	30.4%	27.2%	30.2%
Dyslipidemia [e]	50.6%	51.3%	47.1%	49.7%	49.3%	50.7%
Hypertension [f]	37.6%	39.8%	33.2%	37.3%	31.4%	36.4%
Prediabetes/diabetes [g]	20.7%	21.1%	16.4%	18.3%	14.6%	16.4%
"Ideal" cardiovascular health [h]	8.2%	6.8%	12.6%	8.0%	14.6%	8.2%

[a] Note all estimates are weighted for the survey's complex sampling design. All estimates are age-standardized to the U.S. 2010 population, except for age. Percentage may not sum to 100 due to missing values or rounding. SE= standard error. [b] Insomnia symptoms defined as either trouble falling asleep and/or difficulty maintaining sleep 3+ times a week. [c] Meets PA guidelines defined as ≥150 minutes/week of moderate intensity or ≥75 minutes/week of vigorous intensity or ≥150 minutes/week of moderate and vigorous intensity. [d] Kessler 6-psychological distress scale score ≥13. [e] Dyslipidemia defined as high cholesterol in the 12 months prior to interview. Available for survey years 2011–2017. [f] Hypertension defined as ever told by a doctor had hypertension. [g] Prediabetes/diabetes defined as ever told by a doctor had diabetes or prediabetic condition. [h] "Ideal" cardiovascular health includes never smoking/quit >12 months prior to interview, BMI 18.5 - <25 kg/m^2, meeting physical activity guidelines, and no prior diagnosis of dyslipidemia, hypertension, or diabetes/prediabetes.

Supplemental Table 3. Age-standardized Sociodemographic, Health Behavior, and Clinical Characteristics between Low, Medium, and High Neighborhood Social Cohesion Among Racial/Ethnic Groups, National Health Interview Survey, 2013–2018 (N=167,153) [a].

	**Neighborhood Social Cohesion**											
	**Low *n*=53,364 (32%)**				**Medium *n*=55,163 (33%)**				**High *n*=58,626 (35%)**			
**Sociodemographic**	**NH-White *n*=31,233**	**NH-Black *n*=8,507**	**Hispanic *n*=10,742**	**Asian *n*=2,882**	**NH-White *n*=36,726**	**NH-Black *n*=7,073**	**Hispanic *n*=7,937**	**Asian *n*=3,427**	NH-White *n*=45,173	NH-Black *n*=4,987	Hispanic *n*=5,765	Asian *n*=2,701
Age, mean (S.E.)	46.3 (0.17)	41.8 (0.27)	39.5 (0.23)	42.9 (0.41)	49.1 (0.16)	45.5 (0.29)	41.1 (0.28)	44.7 (0.38)	52.0 (0.15)	48.4 (0.39)	44.8 (0.36)	47.3 (0.48)
18–30 years	18.8%	19.0%	18.4%	17.3%	16.0%	16.2%	17.5%	13.8%	13.4%	16.5%	14.2%	11.7%
31–50 years	20.8%	20.7%	21.3%	22.3%	23.6%	23.4%	22.2%	25.9%	26.2%	23.1%	25.5%	28.0%
≥50 years	60.3%	60.3%	60.3%	60.3%	60.3%	60.3%	60.3%	60.3%	60.3%	60.3%	60.3%	60.3%
Sex/gender												
Women	52.1%	57.5%	53.3%	56.1%	49.2%	53.1%	47.2%	50.7%	52.9%	53.1%	52.6%	55.7%
Educational attainment												
<High school	8.0%	14.2%	34.8%	11.4%	5.5%	11.3%	30.5%	8.5%	5.3%	12.8%	29.9%	7.5%
High school graduate	28.8%	36.2%	28.5%	21.1%	25.4%	32.0%	28.3%	18.4%	25.4%	32.0%	27.9%	18.2%
Some college	33.4%	33.3%	23.9%	20.4%	31.0%	32.2%	25.2%	18.6%	30.8%	30.0%	24.9%	20.1%
≥College	29.8%	16.3%	12.8%	47.2%	38.1%	24.4%	16.0%	54.5%	38.4%	25.2%	17.4%	54.1%
Annual household income												
<$35,000	32.0%	51.9%	46.7%	31.3%	22.0%	41.6%	38.1%	21.7%	19.3%	40.9%	37.8%	21.0%
$35-$74,999	32.5%	29.9%	32.8%	27.0%	29.5%	30.5%	32.9%	25.3%	27.5%	29.9%	30.4%	22.4%
≥$75,000	35.6%	18.2%	20.5%	41.6%	48.5%	27.9%	29.0%	53.0%	53.2%	29.2%	31.8%	56.6%
Unemployed/not in labor force	42.3%	49.0%	43.8%	41.5%	38.0%	44.9%	43.1%	39.3%	38.4%	46.4%	43.4%	38.8%
Occupational class												
Professional/management	20.6%	11.6%	9.0%	26.5%	25.2%	15.2%	12.1%	32.6%	25.0%	14.2%	13.3%	33.2%
Support services	46.3%	44.1%	34.6%	47.3%	46.2%	44.6%	33.9%	43.0%	48.1%	44.2%	36.3%	43.6%
Laborers	33.2%	44.4%	56.3%	26.2%	28.6%	40.2%	54.0%	24.5%	26.9%	41.6%	50.4%	23.2%
Marital/co-habiting status												
Married/living with partner or cohabited	58.2%	37.2%	57.1%	62.7%	65.2%	43.0%	61.0%	69.8%	69.0%	44.2%	63.4%	74.1%
Divorced/widowed	23.2%	29.7%	23.9%	18.9%	18.7%	28.2%	20.9%	15.2%	17.5%	27.7%	20.7%	14.1%
Single/no live-in partner	18.5%	33.1%	19.0%	18.5%	16.1%	28.8%	18.1%	15.0%	13.5%	28.1%	15.9%	11.9%
Region of residence												
Northeast	18.8%	17.4%	15.2%	18.0%	20.0%	15.8%	14.2%	23.4%	19.2%	13.2%	12.3%	17.8%
Midwest	26.8%	18.9%	7.4%	11.8%	27.4%	17.2%	8.7%	10.1%	27.6%	12.2%	8.8%	12.0%

South	32.8%	55.3%	37.9%	20.9%	32.7%	59.0%	36.7%	21.1%	35.2%	68.0%	39.5%	28.3%
West	21.6%	8.4%	39.6%	49.2%	19.9%	8.0%	40.4%	45.3%	18.0%	6.7%	39.4%	42.0%
**Health Behaviors**	**Low**				**Medium**				**High**			
	NH-White	NH-Black	Hispanic	Asian	NH-White	NH-Black	Hispanic	Asian	NH-White	NH-Black	Hispanic	Asian
Sleep duration												
<6 hours (very short)	11.2%	15.4%	11.6%	11.8%	7.4%	11.6%	7.9%	7.9%	6.9%	13.1%	8.1%	7.7%
6–7 hours (short)	24.0%	26.2%	24.1%	26.0%	22.2%	26.4%	21.2%	24.1%	20.0%	23.9%	19.8%	23.6%
7–9 hours (recommended)	60.4%	52.4%	60.2%	58.7%	66.9%	56.7%	67.9%	65.3%	69.4%	56.7%	67.6%	66.4%
>9 hours (long)	4.3%	6.0%	4.1%	3.6%	3.5%	5.4%	3.0%	2.7%	3.6%	6.3%	4.6%	2.4%
Trouble falling asleep (≥3 times/week)	26.9%	23.7%	25.0%	16.5%	19.7%	18.8%	16.8%	12.1%	17.2%	16.2%	16.7%	10.6%
Trouble staying asleep (≥3 times/week)	36.1%	28.3%	25.7%	19.8%	29.4%	23.1%	18.9%	14.5%	26.1%	21.9%	18.9%	12.8%
Insomnia symptoms [b]	43.2%	34.3%	32.9%	25.6%	35.0%	27.8%	24.2%	18.6%	31.3%	25.8%	23.2%	17.0%
Woke up feeling unrested (≥3 days/week)	53.1%	46.2%	48.0%	44.4%	43.8%	40.3%	39.3%	36.7%	37.9%	35.8%	35.1%	30.6%
Sleep medication (≥3 times/week)	13.4%	8.5%	9.5%	4.6%	10.4%	6.5%	6.4%	3.6%	10.1%	6.5%	6.4%	3.1%
Smoking status												
Never/quit >12 months prior[c]	77.0%	80.3%	87.2%	89.0%	82.9%	83.8%	89.6%	91.7%	84.8%	84.3%	89.2%	92.5%
Quit ≤12 months	1.8%	1.1%	0.9%	1.2%	1.5%	1.1%	1.0%	0.5%	1.3%	1.0%	0.9%	0.4%
Current	21.2%	18.5%	11.9%	9.8%	15.5%	15.1%	9.3%	7.8%	13.9%	14.7%	9.9%	7.1%
Alcohol status												
Never	14.0%	29.0%	29.2%	40.9%	13.1%	27.9%	28.8%	38.9%	15.1%	31.2%	31.0%	43.1%
Former	18.1%	18.1%	17.6%	10.7%	14.3%	17.4%	15.7%	9.8%	13.8%	18.0%	14.2%	9.5%
Current	67.9%	52.9%	53.2%	48.4%	72.6%	54.8%	55.5%	51.3%	71.0%	50.8%	54.8%	47.4%
Leisure-time physical activity												
Never/unable	34.5%	45.1%	44.1%	33.0%	27.5%	38.3%	39.0%	27.3%	25.8%	41.0%	39.3%	26.2%
Does not meet PA guidelines	20.0%	18.1%	18.2%	22.0%	19.3%	18.6%	17.0%	22.1%	18.4%	17.1%	15.8%	21.7%
Meets PA guidelines [c]	45.5%	36.8%	37.7%	45.0%	53.2%	43.1%	44.0%	50.6%	55.8%	42.0%	44.8%	52.1%
**Clinical Characteristics**	**Low**				**Medium**				**High**			
	NH-White	NH-Black	Hispanic	Asian	NH-White	NH-Black	Hispanic	Asian	NH-White	NH-Black	Hispanic	Asian

Health status												
Excellent/very good	52.5%	42.9%	43.3%	52.1%	63.0%	48.3%	50.9%	59.7%	68.0%	52.5%	54.2%	64.5%
Good	29.3%	32.1%	32.8%	31.3%	25.8%	32.0%	31.3%	29.7%	22.6%	29.0%	28.8%	25.7%
Fair/poor	18.2%	25.0%	23.9%	16.6%	11.2%	19.6%	17.8%	10.5%	9.4%	18.5%	17.0%	9.8%
Mental illness [d]	6.2%	4.5%	6.5%	2.4%	2.7%	2.7%	2.6%	2.0%	2.1%	3.1%	3.1%	1.2%
Body Mass Index (BMI)												
Recommended BMI (18.5-<25 km/m^2)	32.1%	25.8%	24.8%	55.2%	33.8%	25.7%	25.8%	56.4%	35.5%	24.9%	28.2%	56.9%
Overweight (25–29.9 km/m^2)	33.5%	32.7%	40.3%	32.1%	36.3%	34.6%	41.2%	32.9%	36.7%	33.8%	39.0%	32.5%
Obese (≥30 kg/m^2)	34.5%	41.5%	34.9%	12.7%	29.8%	39.7%	33.0%	10.7%	27.8%	41.3%	32.8%	10.6%
Dyslipidemia [e]	51.6%	49.9%	49.4%	53.4%	47.8%	47.2%	50.6%	56.3%	50.0%	49.2%	50.8%	53.7%
Hypertension [f]	37.6%	48.6%	36.9%	34.1%	37.6%	46.8%	32.8%	31.9%	32.9%	46.8%	33.3%	31.3%
Prediabetes/diabetes [g]	19.0%	24.5%	26.4%	21.0%	15.2%	23.3%	24.1%	19.7%	14.0%	21.9%	21.0%	19.0%
"Ideal" cardiovascular health [h]	8.6%	4.3%	5.4%	12.8%	11.2%	6.3%	7.5%	14.1%	12.6%	5.2%	7.3%	14.9%

[a] Note all estimates are weighted for the survey's complex sampling design. All estimates are age-standardized to the U.S. 2010 population, except for age. Percentage may not sum to 100 due to missing values or rounding. [b] Insomnia symptoms defined as either trouble falling asleep and/or difficulty maintaining sleep 3+ times a week. [c] Meets PA guidelines defined as ≥150 minutes/week of moderate intensity or ≥75 minutes/week of vigorous intensity or ≥150 minutes/week of moderate and vigorous intensity. [d] Kessler 6-psychological distress scale score ≥13. [e] Dyslipidemia defined as high cholesterol in the 12 months prior to interview. Available for survey years 2011–2017. [f] Hypertension defined as ever told by a doctor had hypertension. [g] Prediabetes/diabetes defined as ever told by a doctor had diabetes or prediabetic condition. [h] "Ideal" cardiovascular health includes never smoking/quit >12 months prior to interview, BMI 18.5 - <25 kg/m^2, meeting physical activity guidelines, and no prior diagnosis of dyslipidemia, hypertension, or diabetes/prediabetes.

Supplemental Table 4. Age-standardized Sociodemographic between Neighborhood Social Cohesion Scale. National Health Interview Survey, 2013–2018 (N=167,153) [a]

	**Neighborhood Social Cohesion**			
	**People in this neighborhood help each other out**	**There are people I can count on in this neighborhood**	**People in this neighborhood can be trusted**	**This is a close-knit neighborhood**
All	68,704 (41.1%)	84,914 (50.8%)	78,656 (47.1%)	49,516 (29.6%)
Age				
18–30 years	30.9%	39.6%	36.0%	22.8%
31–50 years	39.0%	47.9%	44.1%	28.3%
≥50 years	45.6%	56.5%	53.8%	32.4%
Sex/gender				
Women	42.4%	52.5%	48.8%	30.4%

Race/ethnicity				
NH-White	45.6%	57.2%	54.6%	31.6%
NH-Black	32.2%	38.5%	30.8%	26.5%
Hispanic/Latinx	31.1%	38.9%	35.1%	25.0%
Asian	38.7%	43.2%	43.2%	28.5%

[a] Note all estimates are weighted for the survey's complex sampling design. All estimates are age-standardized to the U.S. 2010 population, except for age.

Supplemental Figure 1: Flow Chart of Participant Selection.

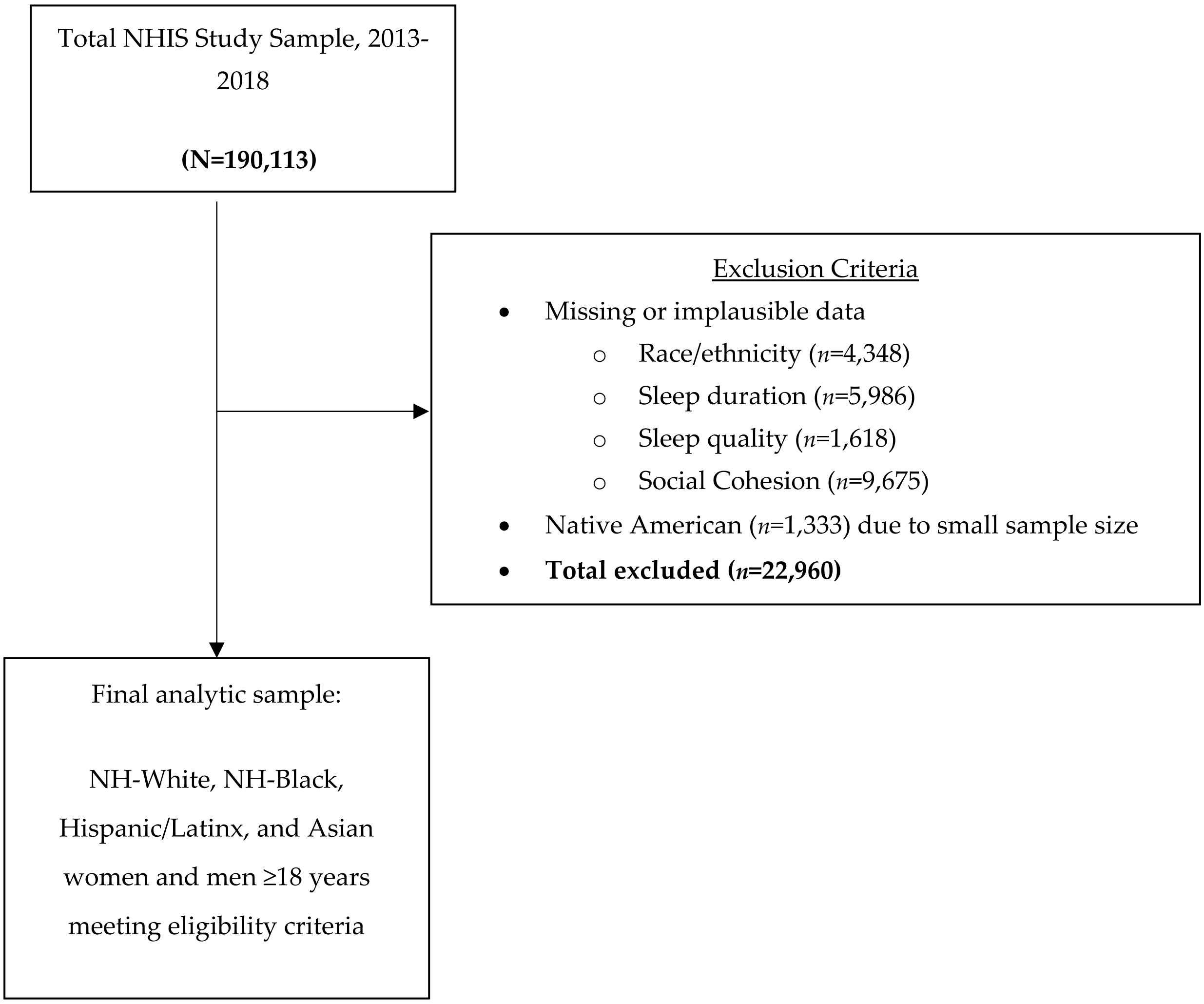

Supplement: Supplementary file 1 [file ijerph-17-09475-s001.pdf]
